# Differential roles of glucosinolates and camalexin at different stages of *Agrobacterium*‐mediated transformation

**DOI:** 10.1111/mpp.12672

**Published:** 2018-04-23

**Authors:** Po‐Yuan Shih, Shu‐Jen Chou, Caroline Müller, Barbara Ann Halkier, Rosalia Deeken, Erh‐Min Lai

**Affiliations:** ^1^ Institute of Plant and Microbial Biology, Academia Sinica 115 Taipei Taiwan; ^2^ Molecular and Biological Agricultural Sciences Program Taiwan International Graduate Program, Academia Sinica 115 Taipei Taiwan; ^3^ Graduate Institute of Biotechnology, National Chung‐Hsing University 402 Taichung Taiwan; ^4^ Chemical Ecology, Bielefeld University 33615 Bielefeld Germany; ^5^ Department of Plant and Environmental Sciences, DynaMo Center University of Copenhagen 1817 Frederiksberg C Denmark; ^6^ Molecular Plant Physiology and Biophysics, Julius‐von‐Sachs‐Institute for Biological Sciences, University of Wuerzburg 97082 Wuerzburg Germany; ^7^ Biotechnology Center, National Chung‐Hsing University 402 Taichung Taiwan

**Keywords:** *Agrobacterium*‐mediated transformation, *Agrobacterium tumefaciens*, camalexin, crown gall, glucosinolates, plant defence, transcriptome

## Abstract

*Agrobacterium tumefaciens* is the causal agent of crown gall disease in a wide range of plants via a unique interkingdom DNA transfer from bacterial cells into the plant genome. *Agrobacterium tumefaciens* is capable of transferring its T‐DNA into different plant parts at different developmental stages for transient and stable transformation. However, the plant genes and mechanisms involved in these transformation processes are not well understood. We used *Arabidopsis thaliana* Col‐0 seedlings to reveal the gene expression profiles at early time points during *Agrobacterium* infection. Common and differentially expressed genes were found in shoots and roots. A gene ontology analysis showed that the glucosinolate (GS) biosynthesis pathway was an enriched common response. Strikingly, several genes involved in indole glucosinolate (iGS) modification and the camalexin biosynthesis pathway were up‐regulated, whereas genes in aliphatic glucosinolate (aGS) biosynthesis were generally down‐regulated, on *Agrobacterium* infection. Thus, we evaluated the impacts of GSs and camalexin during different stages of *Agrobacterium‐*mediated transformation combining *Arabidopsis* mutant studies, metabolite profiling and exogenous applications of various GS hydrolysis products or camalexin. The results suggest that the iGS hydrolysis pathway plays an inhibitory role on transformation efficiency in *Arabidopsis* seedlings at the early infection stage. Later in the *Agrobacterium* infection process, the accumulation of camalexin is a key factor inhibiting tumour development on *Arabidopsis* inflorescence stalks. In conclusion, this study reveals the differential roles of GSs and camalexin at different stages of *Agrobacterium‐*mediated transformation and provides new insights into crown gall disease control and improvement of plant transformation.

## Introduction

The plant pathogen *Agrobacterium tumefaciens* causes crown gall disease by a process initiated when *A. tumefaciens* senses phenolic compounds to induce the expression of virulence genes (Stachel *et al*., 1985). The transferred DNA (T‐DNA) located on the tumour‐inducing (Ti) plasmid is processed and transported from the bacterium into the plant cell. On T‐DNA integration into the plant genome, the expression of T‐DNA‐encoded oncogenes causes the exogenous production of auxin and cytokinin to promote plant cell proliferation and lead to tumour formation. In addition to agrobacterial virulence genes that are required for T‐DNA translocation, a successful transformation also involves several plant host factors (Gelvin, 2010; Gohlke and Deeken, 2014; Hwang *et al*., 2017; Pitzschke, 2013).

Although *A. tumefaciens* seems to be able to hijack host factors to promote T‐DNA transfer and expression for crown gall development, plant defence and hormone response systems are also quickly activated to counteract *Agrobacterium* infection. Microbe‐associated molecular patterns, such as EF‐Tu of *Agrobacterium*, are recognized by the pattern recognition receptor EFR to activate rapid immune responses and compromise *Agrobacterium* infection (Wu *et al*., 2014; Zipfel *et al*., 2006). The activation of basal immune responses is highly regulated by a crosstalk between salicylic acid (SA) and jasmonic acid (JA) (Pieterse *et al*., 2009). *Arabidopsis* SA‐deficient, but not JA‐insensitive, mutants are more susceptible to *Agrobacterium*‐mediated tumour formation, suggesting that SA, but not JA, is important for the control of *Agrobacterium* transformation (Lee *et al*., 2009; Yuan *et al*., 2007). In addition to SA, *Agrobacterium*‐produced auxin (Kutacek and Rovenska, 1991; Lee *et al*., 2009) and cytokinin (Regier and Morris, 1982) also regulate immune responses and transformation efficiency (Hwang *et al*., 2010, 2013; Sardesai *et al*., 2013; Yu and Yang, 1979). These findings suggest that the regulation of plant hormone and immune systems is crucial in the control of *Agrobacterium* transformation efficiency.

Compared with several studies on the role of plant hormones in defence against *Agrobacterium* infection, little is known about plant secondary metabolites in the *Agrobacterium* transformation process. In the Brassicaceae family, to which *Arabidopsis* belongs, glucosinolates (GSs) and the phytoalexin camalexin are key secondary metabolites in defence against pathogen infection. They are synthesized from amino acids, such as tryptophan for indole glucosinolates (iGSs) or camalexin and methionine for aliphatic glucosinolates (aGSs). GSs and their hydrolysis enzymes, the myrosinases, provide a unique defence system which, when plant tissues are damaged, mixes and produces toxic hydrolysis products, typically isothiocyanates (ITCs) and nitriles (Halkier and Gershenzon, 2006). Moreover, the PEN2‐mediated hydrolysis of 4‐methoxyindol‐3‐ylmethyl GS (4MOI3M) serves as a major signal that activates innate immune responses to prevent fungal penetration (Bednarek *et al*., 2009; Clay *et al*., 2009). Camalexin can inhibit the growth of various plant pathogens by disrupting their cell membrane or inducing programmed cell death (Ahuja *et al*., 2012; Rogers *et al*., 1996; Shlezinger *et al*., 2011). Studies on GSs and camalexin deficiency mutants have suggested that both GSs and camalexin are crucial in protecting plants from microbial infection with a broad spectrum of antifungal activity (Bednarek *et al*., 2009; Stotz *et al*., 2011). Their role in bacterial pathogen defence has only been reported for *Pseudomonas syringae* pv. *tomato* DC3000 with a modest effect on bacterial growth *in planta* (Brader *et al*., 2006; Clay *et al*., 2009; Fan *et al*., 2011; Glazebrook and Ausubel, 1994).

Although many studies have characterized the roles of GSs and camalexin in plant defence against herbivores and various pathogens (Bednarek, 2012), to our knowledge nothing is known about their roles in *Agrobacterium*–plant interaction. By transcriptome analysis of *Arabidopsis* seedlings on infection with the virulent *A. tumefaciens* wild‐type strain C58, we noticed that several iGS modifying genes and camalexin biosynthetic genes were up‐regulated, whereas genes in aGS biosynthesis were generally down‐regulated. Similar expression patterns have also been observed in inflorescence stalks on *Agrobacterium* infection. Therefore, in this study, we aimed to elucidate the impact of GS (or rather its hydrolysis products) and camalexin on *Agrobacterium‐*mediated transformation in both seedling and mature plant stages. We discovered that iGS hydrolysis products restrict *Agrobacterium‐*mediated transient transformation at an early infection stage, and that camalexin plays a key role in the negative regulation of later stages of the tumorigenesis process to inhibit tumour development.

## Results

### Time course analysis of *Agrobacterium* infection in *Arabidopsis* seedlings

In order to identify early responsive genes of *Arabidopsis* seedlings to *Agrobacterium* infection, the timing of gene expression of T‐DNA‐encoded genes was monitored in seedlings infected with the virulent *Agrobacterium* wild‐type strain C58 carrying the β‐glucuronidase (GUS) reporter gene (Narasimhulu *et al*., 1996). GUS staining of 7‐day‐old seedlings infected at 2, 8, 24, 48, 72 and 96 h post‐infection (hpi) showed that GUS signals were first detected as early as 48 hpi in both shoots and roots of seedlings and then increased with longer infection time (Fig. S1a–e, see Supporting Information). To determine whether T‐DNA was translocated into plant cells, but not yet expressed prior to 48 hpi, infected *Arabidopsis* seedlings were further recovered in timentin‐containing medium to abolish *Agrobacterium* cell growth, but allow GUS expression. With timentin treatment, GUS signals could be detected in both shoots and roots when seedlings at 24 hpi were recovered for an additional 3 days in timentin‐containing medium (Fig. S1f–j). Our results suggest that T‐DNA translocation into plant cells occurs within 24 hpi, but the accumulation of T‐DNA‐encoded gene products requires more than 1 day to reach detectable levels. Because this study aims to identify early responsive genes to *Agrobacterium* infection, i.e. prior to T‐DNA gene expression, we chose 2 and 24 hpi for transcriptome analysis.

### Common and distinct sets of genes are differentially expressed in shoots and roots of *Arabidopsis* seedlings in response to *Agrobacterium* infection

**Figure 1:**
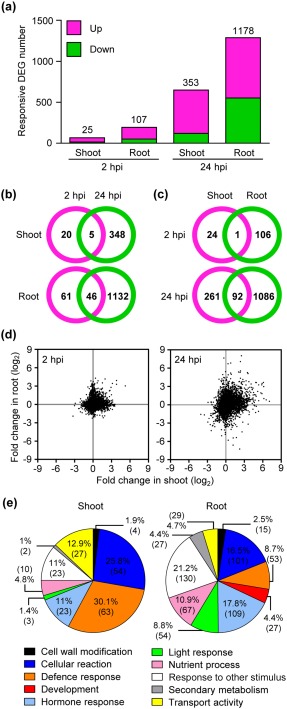
Transcriptional changes in shoots and roots of *Arabidopsis* seedlings in response to *Agrobacterium* infection. Seven‐day‐old *Arabidopsis* Col‐0 seedlings were infected with the virulent *Agrobacterium tumefaciens* strain C58. The differentially expressed genes of shoots and roots of seedlings at 2 and 24 h post‐infection (hpi) were identified by comparing infected seedlings with the mock control from three independent biological replicate samples. (a) The histogram presents the number of up‐regulated (pink) and down‐regulated (green) genes with more than two‐fold changes and Benjamini–Hochberg test correction [false discovery rate (FDR), *P* < 0.05]. Venn diagrams of differentially expressed genes between 2 and 24 hpi in the shoot and root (b) and between shoots and roots at 2 and 24 hpi (c). (d) Scatter plots of whole genome transcriptional changes (log_2_) between tissues at 2 and 24 hpi. The *x*‐axis presents the changes in shoots and the *y*‐axis presents the changes in roots. (e) The distribution of enriched functional gene ontology (GO) groups in shoots and roots at 24 hpi is shown in a pie chart in per cent and number of gene counts in parentheses.

As transient GUS expression signals were stronger and more detectable in shoots (100%) than in roots (no more than 25%) of all infected seedlings (Fig. S1), we separated shoots and roots of infected and non‐infected (mock control) seedlings for gene expression analysis with Affymetrix ATH1 chips. The genes with an intensity higher than the background (value > 75), and with at least two‐fold changes determined by signals of infected plant tissues versus mock controls in all three biological replicates, were classified as differentially expressed genes (DEGs). This analysis resulted in 25 DEGs in shoots (17 up, 8 down) and 107 DEGs in roots (85 up, 22 down) at 2 hpi [false discovery rate (FDR), *P*< 0.05; Fig. 1a and Datasheet S1, see Supporting Information]. At 24 hpi, 353 genes were differentially expressed in shoots (296 up, 57 down) and 1178 in roots (677 up, 501 down) (Fig. 1a and Datasheet S1). Thus, more DEGs were found at 24 hpi than at 2 hpi in both shoots and roots. More than 50% of DEGs in shoots and roots at 2 hpi were not identified at 24 hpi, suggesting that these were only transiently regulated at an early infection stage of *Agrobacterium* infection (Fig. 1b).

The number of DEGs in roots was higher than in shoots (Fig. 1a), although transiently expressed GUS signals were not detected in most roots (Fig. S1e,j). In shoots, more genes were up‐regulated, rather than down‐regulated, whereas, in roots, the number of down‐regulated genes was increased and close to the number of up‐regulated genes (677 up and 501 down at 24 hpi). Strikingly, only one of 25 DEGs (4%) in shoots was shared with DEGs in roots at 2 hpi, whereas 92 of 353 DEGs (26%) in shoots were shared with DEGs in roots at 24 hpi (Fig. 1c). The data suggest a highly tissue‐specific response to *Agrobacterium* infection. In agreement with this notion, scatter plots illustrating whole genome expression patterns showed low correlations between shoots and roots (Fig. 1d).

We identified the majority of DEGs as distinct for shoots and roots. Despite this, a gene ontology (GO) analysis using the 24‐hpi dataset revealed that most enriched GO categories were shared by shoots and roots, except for the development GO, which was only found in roots (Fig. 1e and Tables S1–S4, see Supporting Information). However, the percentage and/or number of gene counts in certain GO categories were significantly different between roots and shoots. For example, both the percentage and number of gene counts in the defence response GO were higher in shoots (30.1%, 63) than in roots (8.7%, 53) at 24 hpi (Fig. 1e and Tables S3 and S4). In contrast, a higher percentage and number of gene counts in the hormone response GO were found in roots (17.8%, 109) than in shoots (11%, 23) (Fig. 1e). In roots, many of the hormone response GO terms related to cytokinin, abscisic acid (ABA), JA and auxin were enriched (Tables S1–S4), with the most significant and abundant GO terms being related to cytokinin. Taken together, these results show that defence responses are regulated more significantly in shoots than in roots, but roots display stronger responses to various hormones than shoots, probably responsible for root growth and development.

### Genes in GS and camalexin biosynthesis are regulated in both *Arabidopsis* seedlings and inflorescence stalks on *Agrobacterium* infection

**Figure 2:**
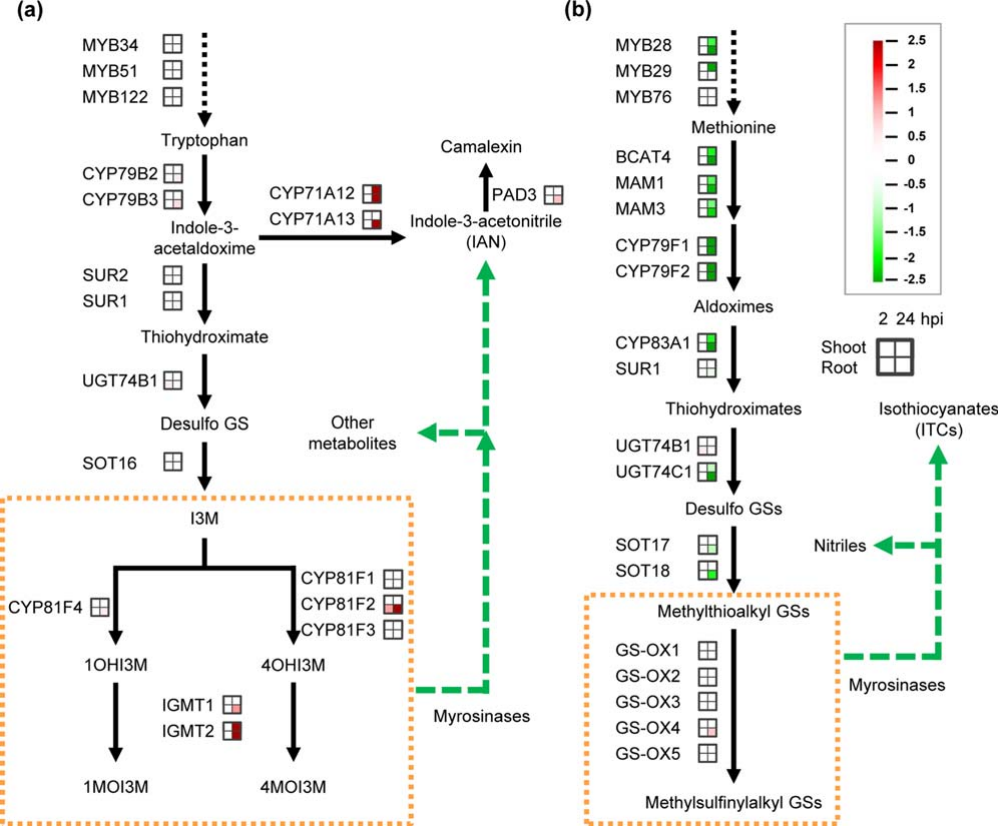
Gene expression profiles of glucosinolate (GS) and camalexin biosynthesis, as well as modification pathways, in response to *Agrobacterium* infection. Indole glucosinolate (iGS) and camalexin (a) and aliphatic glucosinolate (aGS) (b) pathways. The orange squares mark iGSs (a) and aGSs (b). Four squares next to the aGS pathway present the fold changes of key genes in shoots (top) and roots (bottom) at 2 h post‐infection (hpi) (left) and 24 hpi (right). The fold changes are shown in red for up‐regulation and green for down‐regulation as log_2_ values and Benjamini–Hochberg test correction [false discovery rate (FDR), *P* < 0.05]. The expression levels and *P* values of all the genes are also shown in Datasheet S2.

Among the enriched GO terms in the secondary metabolism category, we noticed that GS biosynthesis‐related GO terms were enriched in both shoots and roots (Tables S3 and S4), suggesting a common role of GSs in *Arabidopsis* seedlings in response to *Agrobacterium* infection. Therefore, we analysed the expression profiles of the genes involved in GS biosynthesis in *Agrobacterium*‐infected *Arabidopsis* seedlings, and found that most key genes in iGS modification and camalexin biosynthesis were highly up‐regulated, especially at 24 hpi (Fig. 2a and Datasheet S2, see Supporting Information). The gene encoding CYP81F2, which converts indol‐3‐ylmethyl GS (I3M) to 4‐hydroxyindol‐3‐ylmethyl GS (4OHI3M), was induced at 2 hpi, and further enhanced at 24 hpi, in roots (Fig. 2a and Datasheet S2). The other genes encoding indole glucosinolate methyltransferase 1 and 2 (IGMT1 and IGMT2), converting 4OHI3M or 1OHI3M to 4MOI3M or 1MOI3M, respectively, were also up‐regulated at 24 hpi in roots (IGMT1 and IGM2) and shoots (IGMT2) (Fig. 2a and Datasheet S2). The camalexin biosynthesis genes, including *CYP71A12*, *CYP71A13* and *PAD3*, were highly up‐regulated in both plant parts at 24 hpi, although they were only identified to be statistically significant in roots (*CYP71A12* also in shoots) (Fig. 2a and Datasheet S2). In contrast, most genes involved in aGS biosynthesis were highly suppressed at 24 hpi in roots and shoots (Fig. 2b and Datasheet S2).

Subsequently, we tested whether the observed differential gene expression of GS and camalexin biosynthesis could also be seen in mature plants and at a later stage of infection. According to microarray data (Lee *et al*., 2009), the transcription of genes in camalexin biosynthesis from tryptophan via indole‐3‐aldoxime (IAOx) and indole‐3‐acetonitrile (IAN) was induced at 6 days post‐infection (dpi) and in crown gall tumours, whereas the transcription of genes in iGS biosynthesis from IAOx to I3M was inhibited mainly in tumours (Fig. S2a, see Supporting Information). All genes in aGS biosynthesis were strongly down‐regulated at the later time points, similar to their expression in infected seedlings (Figs 2b and S2b). These findings imply that GS and camalexin may play important roles during *Agrobacterium* infection and may modulate transformation efficiency. Therefore, we aimed to study the impact of GS, camalexin and the derived metabolites on: (i) *Agrobacterium* transient transformation; and (ii) crown gall development.

### iGS hydrolysis plays a negative role in *Agrobacterium* transient transformation efficiency of *Arabidopsis* seedlings

**Figure 3:**
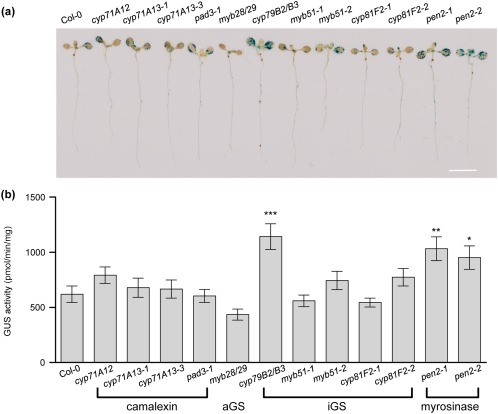
*Agrobacterium* transformation efficiency assay of *Arabidopsis* mutants impaired in glucosinolate and camalexin biosynthesis and in glucosinolate hydrolysis. β‐Glucuronidase (GUS) staining (a) and quantitative GUS activity assay (b) of *Arabidopsis* seedlings after *Agrobacterium* infection at 3 days post‐infection (dpi). All data are presented as the mean ± standard error of the mean (SEM) from four independent experiments (*n* > 20), and asterisks indicate significant changes [one‐way analysis of variance (ANOVA) with Dunnett's test, *P < 0.05, **P < 0.01, ***P < 0.001]. Scale bar, 1 cm. aGS, aliphatic GS; iGS, indole GS.

We first utilized a variety of *Arabidopsis* mutants impaired in iGS, camalexin and aGS biosynthesis to determine their transient transformation efficiencies at the seedling stage. On *Agrobacterium* infection, no statistical difference could be identified in transient GUS activity between Col‐0 and the aGS biosynthesis mutant *myb28/29* (Fig. 3a,b). In contrast, the mutant *cyp79B2/B3*, deficient in iGS and camalexin biosynthesis, showed significantly higher transient GUS activity than Col‐0. Interestingly, the two iGS hydrolysis mutants (*pen2‐1* and *pen2‐2*) were also more susceptible to *Agrobacterium* transformation relative to Col‐0, but there was no detectable difference in transient GUS activity in the camalexin mutants (*cyp71A12*, *cyp71A13‐1*, *cyp71A13‐3* and *pad3‐1*). These results together suggest that the susceptible phenotype detected in the *cyp79B2/B3* mutant should be a result of the depletion of iGS hydrolysis products, but not the depletion of camalexin.

### *Arabidopsis* seedlings reduce the content of specific GSs after *Agrobacterium* infection

**Figure 4:**
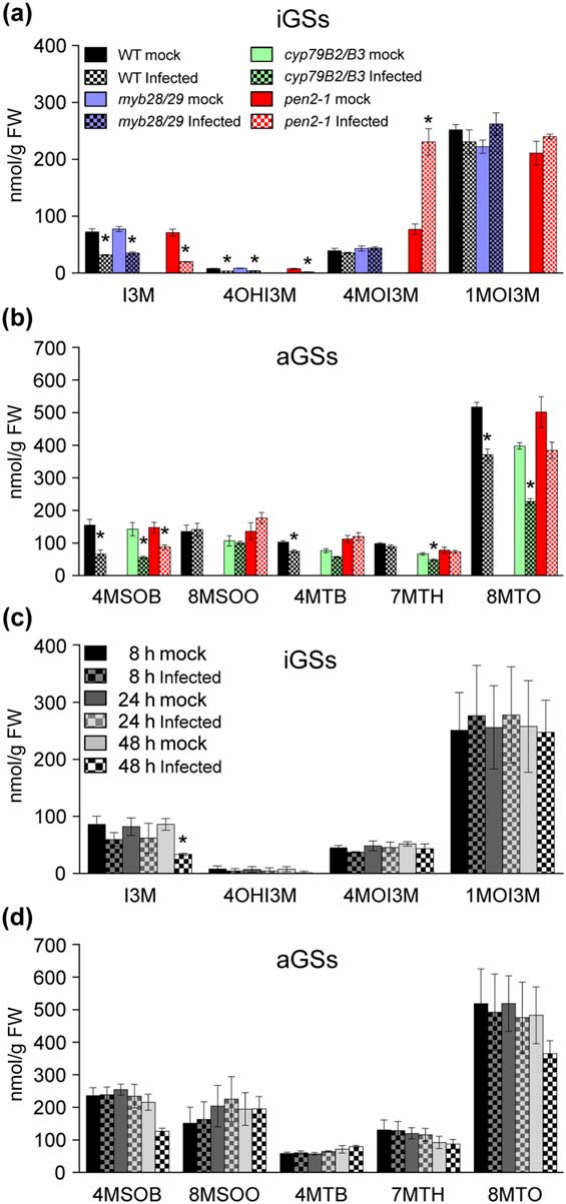
Glucosinolate (GS) profiles of *Agrobacterium*‐infected *Arabidopsis* seedlings. Profiles of indole glucosinolates (iGSs) (a) and the major aliphatic glucosinolates (aGSs) (b) in wild‐type Col‐0 and mutants at 3 days post‐infection (dpi) with *Agrobacterium tumefaciens* strain C58. Time course‐dependent profiles of the iGSs (c) and the major aGSs (d) in Col‐0 seedlings from 8 to 48 h post‐infection (hpi). All data are presented as the mean ± standard error of the mean (SEM) from three independent experiments (*n* = 3), and asterisks indicate significant changes compared with the mock sample (Student's *t*‐test, *P < 0.05). A reduction in (d) of 4‐methylsulfinylbutyl (4MSOB) GS (*P* = 0.051) and 8‐methylthiooctyl (8MTO) GS (*P* = 0.312) at 48 hpi was observed, but is not statistically significant.

To determine the impact of differential gene expression of GS biosynthesis pathways in *Arabidopsis* seedlings on *Agrobacterium* infection, we next analysed the GS profiles of *Arabidopsis* Col‐0 seedlings and selected GS mutants (*myb28/29*, *cyp79B2/B3* and *pen2‐1*) with or without infection at 3 dpi (Figs 4 and S3, and Table S5, see Supporting Information). The concentrations of I3M, 4OHI3M, 4‐methylsulfinylbutyl GS (4MSOB), 4‐methylthiobutyl GS (4MTB) and 8‐methylthiooctyl GS (8MTO), but no other iGSs and aGSs, were significantly reduced in infected Col‐0 seedlings (Figs S3c,d and 4a,b). The most basic iGS, I3M, was significantly reduced from 72 to 32 nmol/g fresh weight (FW) after infection (Fig. 4a). In the secondarily modified iGSs, only 4OHI3M showed a significant reduction in Col‐0 seedlings after infection, but the concentration was too low for it to be considered as a main metabolite (Fig. 4a). No aGSs or iGSs were detected in *myb28/29* or *cyp79B2/B3* mutants, confirming their functions in aGS and iGS biosynthesis, respectively. Moreover, the iGS profile detected in *myb28/29* and the aGS profile detected in *cyp79B2/B3* were similar to those in Col‐0 (Fig. 4a,b). Although the I3M concentration was also significantly reduced in the *pen2‐1* mutant, the 4MOI3M concentration was dramatically increased after *Agrobacterium* infection (Fig. 4a). This observation is consistent with previous reports (Bednarek *et al*., 2009; Clay *et al*., 2009), which suggest that PEN2‐mediated iGS hydrolysis functions in *Agrobacterium*‐infected *Arabidopsis* seedlings.

In *Arabidopsis* seedlings, aGSs are represented by two main groups: the methylthioalkyl GSs and the methylsulfinylalkyl GSs (Fig. 2b and Table S5). We found that two methylsulfinylalkyl GSs, 4MSOB and 8‐methylsulfinyloctyl GS (8MSOO), and three methylthioalkyl GSs, 4MTB, 7‐methylthioheptyl GS (7MTH) and 8MTO, were most abundant in Col‐0 seedlings (Fig. S3d). At 3 dpi, only the concentrations of 4MSOB, 4MTB and 8MTO were decreased, but no significant change was observed for 8MSOO and 7MTH (Figs S3d and 4b). Furthermore, a time course experiment with Col‐0 seedlings showed that most GSs did not change significantly from 8 to 48 hpi, except for I3M, which was significantly reduced at 48 hpi (Fig. 4c,d). We also observed a reduction in 4MSOB and 8MTO concentrations, but the differences were not statistically significant, probably as a result of variations amongst biological replicates (Fig. 4d).

### Exogenous applications of iGS and aGS products impact *Agrobacterium* transient transformation efficiency

**Figure 5:**
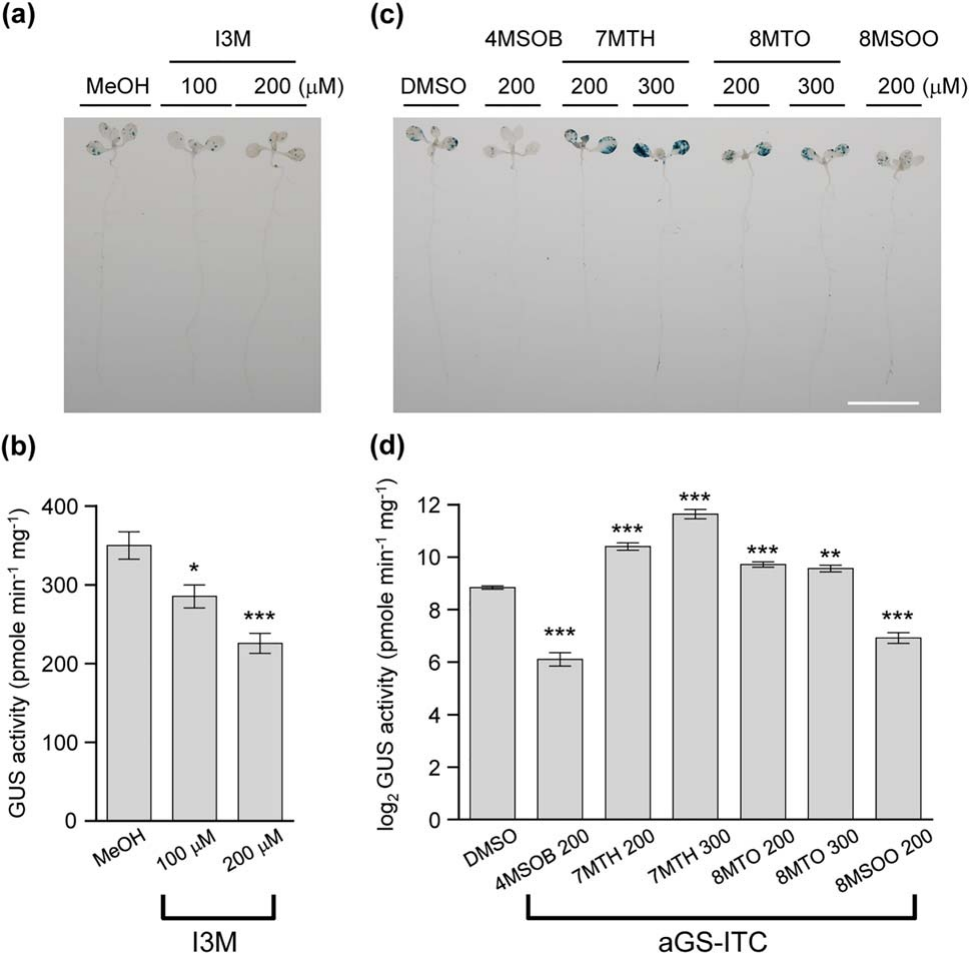
The effects of indole glucosinolate (iGS) and aliphatic glucosinolate‐isothiocyanates (aGS‐ITCs) on *Agrobacterium* transformation efficiency. Seven‐day‐old Col‐0 seedlings were treated with indol‐3‐ylmethylglucosinolate (I3M) (a, b) and aGS‐ITCs (c, d) during *Agrobacterium* infection, followed by analysis for β‐glucuronidase (GUS) staining (a, c) and GUS activity assay (b, d). The results are presented as the mean ± standard error of the mean (SEM) from three independent experiments (*n* ≥ 20), and the GUS activity in (d) is presented as a log_2_ value. Significant differences from the control groups methanol (MeOH) and dimethylsulfoxide (DMSO) are indicated [one‐way analysis of variance (ANOVA) with Dunnett's test, *P < 0.05, **P < 0.01, ***P < 0.001]. Scale bar, 1 cm.

Our GS profile data from *Arabidopsis* seedlings suggest that PEN2‐mediated iGS hydrolysis is activated in *Arabidopsis* seedlings on *Agrobacterium* infection (Fig. 4), which is consistent with a negative role of PEN2 myrosinase in the transient transformation process (Fig. 3). Thus, we next directly treated the seedlings with I3M during *Agrobacterium* infection, and found that the transient transformation efficiency was reduced in a dose‐dependent manner relative to methanol‐treated control seedlings (Fig. 5a,b). As exogenous application of I3M does not influence the number of viable *Agrobacterium* cells grown in either co‐cultivation medium or associated with infected seedlings at 1 or 3 dpi (Figs S5a,b, see Supporting Information) and has no impact on the GUS enzymatic activity *in vitro* (Fig. S4a, see Supporting Information), we suggest that the reduced transient transformation efficiency on I3M application is probably caused by its impact on the host plant.

Although we did not observe a clear statistical difference in the transient transformation efficiencies between the *myb28/29* mutant and Col‐0, the mutant showed a tendency to a lower transient transformation efficiency in general (Fig. 3b). Thus, we tested whether the most prevalent hydrolysis products from aGSs, the ITCs (Wittstock and Burow, 2010), may have a direct impact on *Agrobacterium‐*mediated transient transformation efficiencies. We tested the effect of ITCs derived from 4MSOB, 7MTH, 8MTO and 8MSOO on the transient transformation efficiency of Col‐0 seedlings by the application of ITCs in the co‐cultivation medium at 200 and 300 μm. The results showed that application of the aliphatic ITCs resulted in different effects, with the transient transformation efficiency being inhibited when the seedlings were treated with ITCs of 4MSOB (85% reduction) or 8MSOO (74% reduction) (Fig. 5c,d), and enhanced when the seedlings were treated with ITCs of 7MTH (∼3–7‐fold increase) and 8MTO (>1.5‐fold increase). It is notable that exogenous application of these aGS‐ITCs caused 10%–20% reduction in GUS enzymatic activity *in vitro* (Fig. S4b). Thus, we conclude that 7MTH and 8MTO indeed play positive roles in promoting transient transformation efficiency, despite their modest effects in inhibiting GUS enzymatic activity *per se*. However, the reduced transient GUS expression of infected seedlings treated with 4MSOB‐ITC and 8MSOO‐ITC may be partly caused by their direct effects in inhibiting GUS enzymatic activity.

### Impact of exogenous applications of aGS products on A*grobacterium* growth

To determine whether the different impacts of aGS‐ITCs on transformation efficiency may contribute to differences in *Agrobacterium* growth during co‐cultivation, we measured the viable *Agrobacterium* cell number in both the co‐cultivation medium and cells associated with seedlings at 1 and 3 dpi. The aGS‐ITC treatments did not affect the viable *Agrobacterium* cell number in the co‐cultivation medium at either 1 or 3 dpi, except for 4MSOB, which caused a modest reduction in viable cell number at 1 dpi (Fig. S5d). However, no difference in the seedling‐associated *Agrobacterium* cell number was observed for seedlings treated with 4MSOB or dimethylsulfoxide (DMSO) control at 1 or 3 dpi (Fig. S5c), suggesting that the reduced transient transformation efficiency of the Col‐0 seedlings treated with 4MSOB (Fig. 5c,d) could not be attributed to the number of *Agrobacterium* cells. Similarly, the reduced transient transformation efficiency of the Col‐0 seedlings treated with 8MSOO (Fig. 5c,d) is probably not impacted by *Agrobacterium* growth, as no reduced *Agrobacterium* cell number was observed in the medium or *in planta* in the presence of 8MSOO (Fig. S5c,d). However, the seedling‐associated *Agrobacterium* cells were significantly higher in number when treated with 7MTH‐ITC or 8MTO‐ITC than mock control at 1 and 3 dpi when a higher concentration (300 μm) was applied (Fig. S5c,d). It is notable that more viable *Agrobacterium* cells were only observed *in planta*, but not in co‐cultivation medium, which contains one to two orders more viable *Agrobacterium* cells than those *in planta*. Thus, 7MTH‐ITC and 8MTO‐ITC do not have an impact on *Agrobacterium* growth, but can facilitate *Agrobacterium* association with Col‐0 seedlings and enhance transformation efficiencies.

### Camalexin, but not GSs, contribute to *Agrobacterium*‐mediated tumour formation on *Arabidopsis* inflorescence stalks

**Figure 6:**
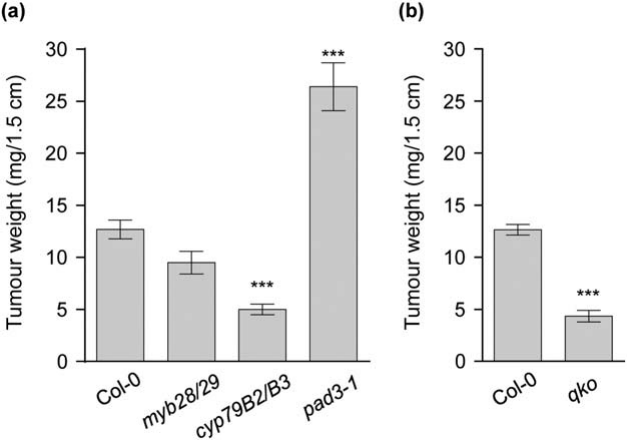
Tumorigenesis assay on inflorescence stalks of *Arabidopsis* Col‐0 and mutants lacking either camalexin and/or glucosinolates (GS). (a) The tumour weight of the biosynthesis mutants for aliphatic GS (*myb28/29*), indole GS (*cyp79B2/B3*) and camalexin (*pad3‐1*). (b) The tumour weight of the GS‐free mutants (*qko*). The mean fresh weight ± standard error of the mean (SEM) of crown galls was calculated of three to six independent experiments with a minimum of 11 plants per experiment. Significant differences from the control plants are indicated [one‐way analysis of variance (ANOVA) with Dunnett's test, ***P < 0.001].

We next determined whether GS and camalexin biosynthesis pathways also impact tumorigenesis, the later stage of infection. Surprisingly, tumours of *cyp79B2/cyp79B3* were significantly smaller than, and those on *myb28/myb29* showed no difference from, those of wild‐type Col‐0 at 4 weeks after inoculation of young inflorescence stalks (Fig. 6a). The *pad3‐1* mutant developed significantly larger crown galls relative to the wild‐type. These results differed from the transient transformation efficiencies detected in *cyp79B2/cyp79B3* (increased) and *pad3‐1* (unchanged; Fig. 3). The quadruple mutant *qko* (*cyp79B2/cyp79B3/myb28/myb29*) (devoid of GSs and camalexin) developed very small tumours or none at all (Fig. 6b), which is similar to *cyp79B2/cyp79B3*. Because the tumorigenesis efficiency is a result of the combination of cell proliferation ability and transformation efficiency, including steps of T‐DNA translocation from the bacterium into plant cells, T‐DNA trafficking from the cytoplasm into the nucleus, T‐DNA integration and T‐DNA gene expression, we tested the callus induction ability of these mutants using *Arabidopsis* root explants following the methods described previously (Hwang and Gelvin, 2004). We did not observe any significant difference between Col‐0 and the tested mutants including *cyp79B2/B3*, *myb28/29* and *pad3‐1* (Fig. S6a, see Supporting Information). Thus, cell proliferation ability is unlikely to be the key reason for the reduced tumour size of the *cyp79B2/cyp79B3* mutant and the increased crown gall development of *pad3‐1*.

**Figure 7:**
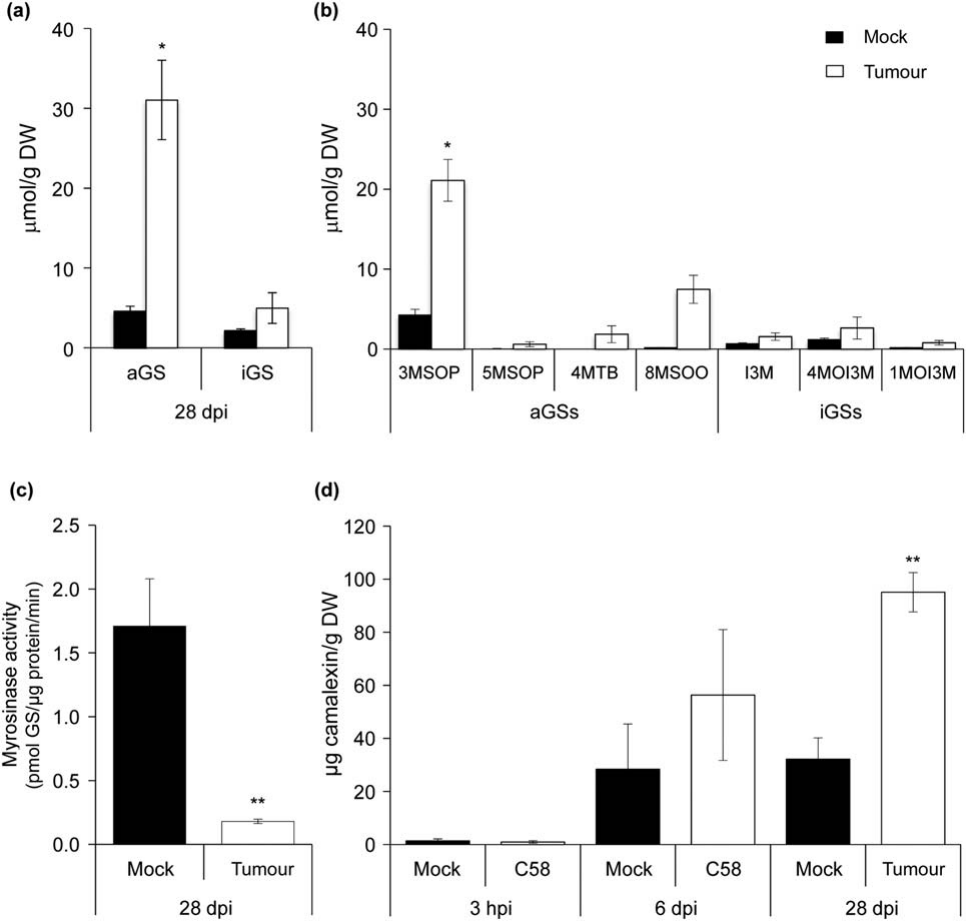
Glucosinolate (GS) and camalexin content in crown galls. Mean amounts of total aliphatic GSs (aGSs) and indole GSs (iGSs) (a) and the main aGSs and iGSs (b) in crown galls and mock‐inoculated inflorescence stalk tissue. (c) Mean myrosinase enzyme activity in crown galls and mock‐inoculated inflorescence stalks of three independent biological replicates with two samples in each experiment. (d) Camalexin content of *Arabidopsis* inflorescence stalks inoculated with *Agrobacterium* or mock‐inoculated at three different time points. The mean values of three to four independent biological replicates ± standard error of the mean (SEM) are shown. Each replicate consisted of material from more than 10 individual plants. dpi, days post‐infection; DW, dry weight; hpi, hours post‐infection.

We found a 6.9‐fold higher concentration of aGSs in tumours than in mock‐inoculated inflorescence stalks at 28 dpi, but no significant difference for iGSs (Fig. 7a). In tumours, the major aGSs were 3‐methylsulfinylpropyl GS (3MSOP), 5‐methylsulfinylpentyl GS (5MSOP), 4MTB and 8MSOO, whereas I3M, 4MOI3M and 1MOI3M were the major iGSs (Fig. 7b). The myrosinase enzyme activity was significantly lower in crown galls than in mock‐inoculated tissues (Fig. 7c), which suggests that crown gall tissues may not activate GS hydrolysis like non‐inoculated plant tissues.

### Camalexin accumulates after *Agrobacterium* infection and inhibits transient transformation in *Arabidopsis* seedlings when exogenously applied

**Figure 8:**
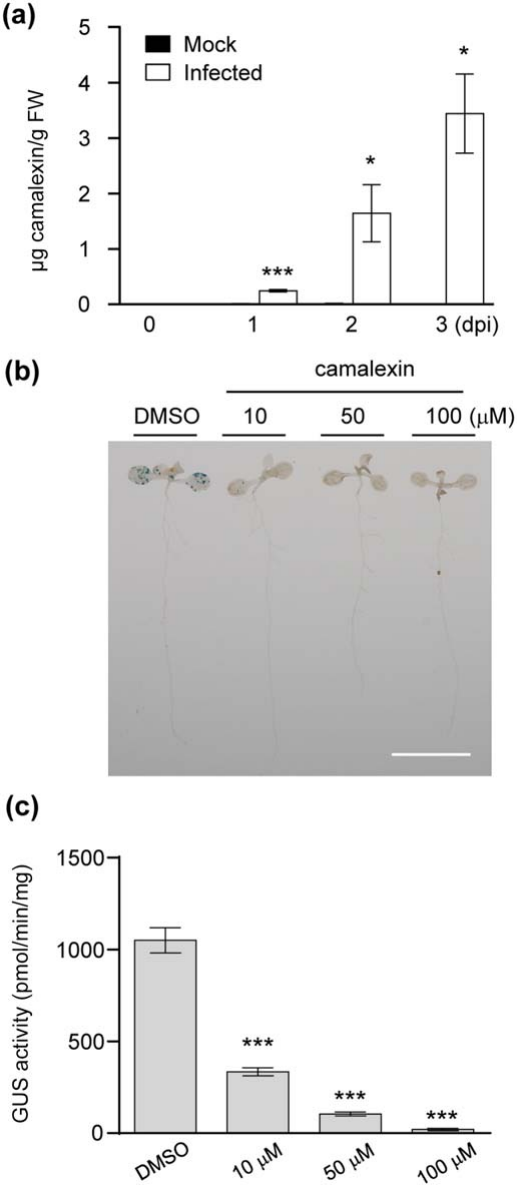
The effect of camalexin in *Agrobacterium*‐mediated transformation of *Arabidopsis* seedlings. (a) Camalexin quantification in mock or infected wild‐type Col‐0 seedlings from 0 to 3 days post‐infection (dpi). Only the mock group was measured at 0 dpi. β‐Glucuronidase (GUS) staining (b) and GUS activity assay (c) for transient transformation after treatment with different concentrations of camalexin for 3 days. The results are presented as the mean ± standard error of the mean (SEM) from three independent experiments (*n* ≥ 15), and significant differences from the mock infection group (a, Student's *t*‐test, *P < 0.05, ***P < 0.001) or from the control dimethylsulfoxide (DMSO) group [b and c, one‐way analysis of variance (ANOVA) with Dunnett's test, *** *P* < 0.001] are indicated. Scale bar, 1 cm.

Camalexin is a phytoalexin that inhibits the growth of various plant pathogens and often negatively impacts pathogen infection (Ahuja *et al*., 2012; Rogers *et al*., 1996). It is intriguing to note that the *pad3‐1* mutant exhibited similar transient transformation efficiency to the wild‐type during the seedling stage, but was highly susceptible to tumorigenesis (Figs 3 and 6a). One plausible reason is that the camalexin concentration may be different during different developmental stages. Thus, we measured the camalexin concentrations in *Arabidopsis* seedlings during the course of *Agrobacterium* infection and at later stages of tumorigenesis. Camalexin could not be detected in seedlings before infection (0 dpi) and the concentration gradually increased during the infection time, with 0.24 μg/g FW (1.2 μm) at 1 dpi, 1.64 μg/g FW (8.2 μm) at 2 dpi and 3.44 μg/g FW (17.2 μm) at 3 dpi (Fig. 8a), and correlated with the gene expression profile (Fig. 2a and Datasheet S2). In crown galls, the camalexin concentration was also lower at 3 hpi, but significantly higher at 6 dpi and further increased at 28 dpi (Fig. 7d).

We then treated the Col‐0 seedlings with increasing concentrations of camalexin in co‐cultivation medium during *Agrobacterium* infection to determine the impact of camalexin on the transient transformation efficiency during the seedling stage. The GUS signal was dramatically reduced in seedlings treated with 10 μm camalexin, and almost no transiently expressed GUS activity could be detected on application of 100 μm camalexin (Fig. 8b,c). As the exogenous application of camalexin did not affect or only slightly inhibited GUS enzymatic activity *in vitro* with higher dosage (Fig. S4c), the negative impact of camalexin on transient GUS expression in infected seedlings is probably the result of reduced transient transformation efficiency rather than affecting GUS enzymatic activity *per se*. Because the application of camalexin did not inhibit *Agrobacterium* cell growth in co‐cultivation medium or associated with infected seedlings at 1 or 3 dpi (Fig. S5e,f), the *Agrobacterium* cell number has no impact on the reduced transient transformation efficiency of camalexin‐treated seedlings. However, although no difference in callus induction ability of root explants could be detected with the camalexin‐deficient mutant *pad3‐1*, exogenous application of camalexin can reduce the callus induction rate of root explants and transient transformation efficiencies of seedlings, both in a dose‐dependent manner (Figs 8c and Fig. S6b). These results suggest that camalexin exhibits negative effects on both transformation efficiency and cell proliferation when the camalexin concentration reaches the threshold of ∼10 μm, which can be reached in seedlings at 2 dpi (Fig. 8a) and in wounded/infected inflorescence stalks at 6 dpi (Fig. 7d). Taken together, these results suggest that camalexin plays a negative role in both the early stage of transformation and the later stage of tumorigenesis, which may be partly a result of the reduced cell proliferation activity of plant cells when the camalexin concentration is high.

## Discussion

In this study, the transcriptome analysis of *Agrobacterium‐*infected seedlings and crown galls of *Arabidopsis* plants led to the discovery of DEGs involved in GS and camalexin biosynthesis pathways during the *Agrobacterium* infection process. Furthermore, functional studies suggested a potential role of iGS hydrolysis products in seedling defence against *Agrobacterium* infection at an early stage, whereas camalexin plays a key role in the inhibition of tumour development at a later stage of the *Agrobacterium* transformation process.

Although the GS‐related GO terms were enriched in both shoots and roots of seedlings at 24 hpi (Tables S3 and S4), the biosynthesis of iGSs and aGSs was differentially regulated. The induction of genes in I3M modification (CYP81F2, IGMT1 and IGMT2 which convert I3M into 4MOI3M) as early as 2 hpi (Fig. 2a and Datasheet S2) was correlated with a reduced content of I3M at later stages of infection (2 and 3 dpi; Fig. 4a,c). Previous studies have suggested that the reduction in I3M is an early response to pathogen infection, and that PEN2‐mediated iGS hydrolysis provides defence signalling (Bednarek *et al*., 2009; Clay *et al*., 2009). This is also supported by our findings of higher transient transformation efficiencies of *Arabidopsis* mutant seedlings deficient in iGS biosynthesis and hydrolysis (Fig. 3). Furthermore, the transient transformation efficiency was decreased when Col‐0 seedlings were treated with I3M (Fig. 5a,b). I3M did not inhibit agrobacterial growth in co‐cultivation medium or *in planta* (Fig. S5a,b), and an *in vitro* study has shown that *Agrobacterium* is more resistant to iGS than to aGS hydrolysis products (Aires *et al*., 2009). Thus, the reduction in transformation efficiency by iGS was not a result of the restriction of agrobacterial growth, but probably caused by the immune response triggered by iGS hydrolysis. However, we do not exclude the possibility that iGS hydrolysis may also impact other physiological functions or signal transduction of *A. tumefaciens* and its infected plant cells, which may then affect the transient transformation efficiency. Such studies are beyond the scope of this work and await future investigations.

However, the *myb28/29* mutant (which lacks aGSs) did not show a statistically significant difference in either transient transformation efficiency or tumour development (Figs 3 and 6a). Beekwilder *et al*. (2008) have shown higher iGS concentrations in this mutant than in Col‐0 after insect attack, but such an increase was not found in our seedling assay systems, as *myb28/29* mutant seedlings contained no aGSs and similar iGS amounts to wild‐type Col‐0 (Fig. 4a). Thus, we suggest that iGSs do not have a negative impact on the transformation efficiency of the *myb28/29* mutant.

To this end, the biological significance of the reduced expression of aGS biosynthetic genes in *Arabidopsis* seedlings after *Agrobacterium* infection and during crown gall development remains unclear. The global reduction in gene expression of aGS biosynthesis was reflected in the reduction in certain aGS amounts at 3 dpi (Figs 2b and 4b,d). The aGSs with side chains of four carbon atoms (4C; 4MTB and 4MSOB), 7C (7MTH) and 8C (8MTO and 8MSOO) were most abundant in 7–10‐day‐old *Arabidopsis* seedlings (Fig. S3d). However, only 4MSOB, 4MTB and 8MTO were significantly reduced after *Agrobacterium* infection (Fig. 4b), which suggests a selective regulation of 4C and 8C aGS biosynthesis.

It is interesting to note that exogenous applications of different aliphatic ITCs caused different impacts on the *Agrobacterium* transformation efficiencies in *Arabidopsis* seedlings. The transient transformation efficiencies were inhibited by exogenous applications of 4MSOB‐ITC and 8MSOO‐ITC, but enhanced with 7MTH‐ITC and 8MTO‐ITC treatments (Fig. 5c,d). Although 7MTH‐ITC and 8MTO‐ITC caused about 10% reduction in the GUS enzymatic activity *per se* (Fig. S4b), exogenous applications of 7MTH and 8MTO can still enhance transient GUS expression of infected seedlings. Thus, 7MTH and 8MTO indeed play positive roles in the promotion of transient transformation efficiency. However, 4MSOB‐ITC and 8MSOO‐ITC caused about 10%–20% reduction in GUS enzymatic activity *in vitro* (Fig. S4b), but more than 70% reduction in transformation efficiency, as reflected by the transient GUS expression levels. This indicates that 4MSOB‐ITC and 8MSOO‐ITC play a negative role in transient transformation, and the reduced transient GUS expression of infected seedlings may be partly a result of their negative influence on GUS enzymatic activity.

ITCs are known to be toxic to various pathogens, and a previous *in vitro* assay has shown that 4MSOB‐ITC inhibits *Agrobacterium* cell growth (Aires *et al*., 2009). Consistent with this finding, our results also showed that 4MSOB‐ITC caused a modest reduction in viable cell number at 1 dpi (Fig. S5d). However, all other tested ITC compounds did not affect *Agrobacterium* growth in co‐cultivation medium (Fig. S5c,d). Interestingly, the presence of 7MTH‐ITC and 8MTO‐ITC facilitated *Agrobacterium* association to Col‐0 seedlings and enhanced transformation efficiencies, even though it did not affect *Agrobacterium* cell growth in co‐cultivation medium. Because several *Arabidopsis* mutants with enhanced defence responses showed reduced *Agrobacterium* binding to their root explants (Gaspar *et al*., 2004; Sardesai *et al*., 2013), we propose that 7MTH‐ITC and 8MTO‐ITC suppress the defence responses of Col‐0 seedlings, which then become more susceptible to *Agrobacterium* attachment, thereby promoting transformation efficiency.

Unlike the observations in *Arabidopsis* at an early *Agrobacterium* infection stage, where the total amounts of iGS and aGS were not increased relative to the mock control, crown galls hyperaccumulated particularly the aGSs 3MSOP and 8MSOO. In contrast, the content of iGSs was low and similar to the concentrations in mock‐infected inflorescence stalks (Fig. 7a,b). Because all genes in aGS biosynthesis were suppressed during tumour development (Fig. S2b), the accumulation of aGSs in crown galls probably comes from long‐distance transport via the *Arabidopsis* phloem (Chen *et al*., 2001). A similar observation was reported for *Arabidopsis* phloem‐feeding and chewing insects, which specifically caused a significant increase in the amounts of aGSs, but not iGSs (Yan and Chen, 2007). The accumulated aGSs are probably not degraded because of low myrosinase enzyme activity in crown galls (Fig. 7c) and may not play a major role at the tumour stage. However, *cyp79B2/B3* and *qko* (quadruple *cyp79B2/cyp79B3/myb28/myb29* mutant which lacks iGSs and camalexin) showed significantly reduced tumour size (Fig. 6a,b). Their similar tumour sizes further support the conclusion that aGSs do not play a major role at the tumour stage.

It remains unclear why the *cyp79B2/B3* mutant was more susceptible to transient transformation, but developed smaller tumours. Plant cell proliferation may not be a problem in this mutant because it showed similar callus induction rates relative to Col‐0 (Fig. S6a). Previous reports have shown that *cyp79B2/B3* double mutant plants contain wild‐type levels of indole‐3‐acetic acid (IAA) and grow in a similar manner to the wild‐type under normal growth conditions (Stepanova *et al*., 2011; Zhao *et al*., 2002), which is consistent with our observation of no detectable growth phenotype in this mutant. The reduced IAA contents and growth phenotype in the *cyp79B2/B3* mutant are temperature and light dependent, and can be restored to wild‐type levels by supplying the IAA precursor indole‐3‐acetamide (IAM) (Sugawara *et al*., 2009; Zhao *et al*., 2002). This suggests that the auxin signalling and biosynthesis pathways remain active in the *cyp79B2/B3* mutant. It is known that modifications in the biosynthetic pathway to iGS affects auxin production, because more precursors are available for IAA biosynthesis when the iGS pathway is interrupted (Malka and Cheng, 2017). In *Arabidopsis* crown galls, IAA can be produced via the agrobacterial enzymes IaaM and IaaH, in addition to plant‐specific pathways (Britton *et al*., 2008). Thus, in crown galls of the *cyp79b2/b3* double mutant, IAA may be overproduced and cause growth inhibition of the tumour because the iGS pathway is interrupted. An example for this hypothesis is the *sur1* mutant, which produces less iGS, but increasing amounts of IAA, which causes growth inhibition of *sur1* (Boerjan *et al*., 1995; Malka and Cheng, 2017). Calli induced in *cyp79B2/B3* do not express the agrobacterial IAA biosynthesis pathway; therefore, IAA is only produced by the plant‐specific pathway, does not over‐accumulate and therefore callus growth is wild‐type‐like. Therefore, we propose that the mis‐regulation of endogenous free IAA could be a possible reason for the small tumour in the *cyp79B2/B3* mutant, although a possible role of iGS or some indole‐3‐acetaldoxime (IAOx)‐derived metabolite in promoting tumour formation cannot be excluded.

However, despite the finding that the camalexin biosynthesis genes *CYP71A12*, *CYP71A13* and *PAD3* were up‐regulated at 24 hpi, no significant change in the transient transformation efficiency could be observed in these mutants relative to Col‐0 (Fig. 3), probably because of the low levels of camalexin produced in seedlings at the early stage of infection (Fig. 8a). However, Col‐0 seedlings showed reduced transient transformation efficiency when camalexin was present in the co‐cultivation medium at 10 μm (Fig. 8b,c), the concentration neither influencing GUS activity *per se* (Fig. S4c) nor inhibiting bacterial growth in medium or the association with seedlings (Figs S5e and 5f). Interestingly, exogenous application of camalexin can also reduce the callus induction ability of Col‐0 root explants at 10 μm or higher in a dose‐dependent manner (Figs 8c and S6b). Thus, we suggest that larger tumour development in the *pad3‐1* mutant (Fig. 6a) is caused by both higher T‐DNA transformation efficiency at an early stage and increased cell proliferation at a later stage of tumour development because of the lack of camalexin.

In conclusion, this study provides transcriptomic data revealing both common and distinct genes expressed in shoots and roots of *Arabidopsis* seedlings in response to *Agrobacterium* infection. Focusing on the roles of GS and camalexin, we showed that iGS hydrolysis plays a defensive role at the early stage of *Agrobacterium* infection and causes reduced transient transformation efficiency, whereas camalexin accumulates during tumour development to restrict tumorigenesis. The findings that I3M and camalexin inhibit, and that 7MTH‐ITC and 8MTO‐ITC promote, transient transformation efficiency in *Arabidopsis* seedlings may be applicable to crown gall control and improve *Agrobacterium‐*mediated transformation efficiency, respectively. Because GSs and camalexin are found mainly in certain Brassicaceae species, it remains to be tested whether these compounds can function in other crop species. It is worth studying the underlying mechanisms of these chemicals in regulating *Agrobacterium* transformation.

## Experimental Procedures

### Plant materials

*Arabidopsis thaliana* ecotype Col‐0 was used for the seedling transformation assay, transcriptome assay and *Agrobacterium* inoculation assay. GS mutants in the Col‐0 background, including *myb28/myb29* (SALK_136312 x GABI_868E02), *cyp81F2‐1* (SALK_073776), *cyp81F2‐2* (SALK_123882), *myb51‐1* (SM_3_16332), *myb51‐2* (SALK_059765), *cyp79B2/cyp79B3* (Zhao *et al*., 2002), *pen2‐1* (Lipka *et al*., 2005) and *pen2‐2* (GABI‐KAT 134C04), the camalexin mutants, including *cyp71A12* (GABI‐KAT 127‐H03), *cyp71A13‐1* (SALK_105136), *cyp71A13‐3* (SALK_128994) and *pad3‐1* (CS3805), and the *cyp79b2/B3/myb28/29* quadruple (qko) mutant, completely free of GSs and camalexin, were used in the transient seedling transformation assay as described.

### *Agrobacterium* transformation of *Arabidopsis* seedlings and GUS assays

The virulent *A. tumefaciens* wild‐type strain C58 was used for the infection of Col‐0 seedlings. Seeds were germinated in 2 mL of half‐strength Murashige and Skoog (MS) (Basal Salt Mixture, PhytoTechnology Laboratories, Kansas City, Kansas, USA) liquid medium [half‐strength MS salt supplemented with 0.5% sucrose (w/v), pH 5.7] in each well of a six‐well plate. Germination and growth took place in a growth room at 22 °C under a 16‐h/8‐h light–dark cycle (100 µmol/m^2^/s). Virulence of *A. tumefaciens* was pre‐induced by 200 µm acetosyringone in AB‐MES (AB Minimal Medium plus MES salt, pH 5.5) (Wu *et al*., 2014) at 25 °C for 16 h prior to infection. The *Arabidopsis* seedlings were infected with pre‐induced *A. tumefaciens* C58 cells at an optical density at 600 nm (OD_600_) = 0.02 in half‐strength MS medium. If the removal of agrobacterial cells was necessary, co‐cultivation medium was removed after the chosen infection time and replaced with 2 mL of freshly prepared half‐strength MS medium containing 100 µm timentin, and incubated for recovery before analysis.

For the monitoring of the transient transformation efficiency, the T‐DNA vector pBISN1 carrying the *gusA‐intron* genes (Narasimhulu *et al*., 1996) was transformed into *A. tumefaciens* strain C58 for infection of *Arabidopsis* seedlings. GUS staining and activity assays were carried out as described at the chosen infection time (Salinas and Sánchez‐Serrano, 2006; Wu *et al*., 2014). In brief, seedlings were stained by incubation in GUS staining solution containing 5‐bromo‐4‐chloro‐3‐indolyl glucuronide (X‐Gluc), and incubated at 37 °C in the dark overnight, followed by destaining in 90% ethanol (EtOH). For the GUS activity assay, liquid nitrogen‐frozen seedlings from each well were ground into a fine powder to extract total protein. The GUS activity in 20 µg of protein per 200‐μL reaction was quantified with the fluorescence substrate 4‐methylumbelliferyl‐β‐d‐glucuronide (MUG). The fluorescence intensity (excitation, 365 nm; emission, 455 nm; filter at 430 nm) was measured using a Microplate Reader (BioTek, Taipei, Taiwan) at 37 °C for 1 h. GUS activity was normalized to the protein amount and 4‐methylumbelliferone standard curve. For statistical analysis, one‐way analysis of variance (ANOVA) with Dunnett's test was performed. To determine the effects of GS‐derived metabolites and camalexin on GUS enzyme activity *in vitro*, the selected compounds and DMSO control were each incubated with 5 ng of recombinant GUS protein (Sigma‐Aldrich, St. Louis, MO, USA) in GUS extraction buffer containing 1 mm MUG. The reaction mixture was measured for GUS activity at 37 °C for 1 h.

### Transcriptome analysis

For gene expression profiling of *Agrobacterium*‐infected seedlings, the shoots and roots of Col‐0 seedlings (infected or mock control) were separated by cutting with a micro‐scissor and immediately frozen in liquid nitrogen. Total RNA was extracted according to the phenol (pH 4.5)/chloroform protocol, followed by gene expression analysis with Affymetrix ATH1 chips (Affymetrix, Santa Clara, CA, USA). The chips of three biological repeats were normalized by the MAS5.0 algorithm using GeneSpring software (Agilent Technologies, Santa Clara, CA, USA), and the genes with an intensity higher than the background value (value > 75), which passed the asymptotic unpaired *t*‐test with Benjamini–Hochberg test correction (FDR, *P* < 0.05), were selected for further analysis. The fold changes were determined from the signals of infected plant tissues versus mock infection controls under the same conditions, and two‐fold changes were used as cut‐off to determine *Agrobacterium*‐responsive genes. GOBU software (Lin *et al*., 2006) was used to analyse GO. The significant GO items were calculated with elim Fisher's exact test (*P* < 0.01) based on gene counts (Alexa *et al*., 2006).

### Crown gall growth assay

For the crown gall growth assay, the *A. thaliana* wild‐type Col‐0 and mutants were cultivated in growth cabinets (Percival, CLF, Wertingen, Germany) under short‐day conditions at 22 °C (8 h of 80–100 µmol/m^2^/s light; Osram 400 W, Power Star HQI‐E 400W/DV, 380–780 nm) (Wuerzburg, Germany) and 16 °C during the dark period (16 h) with a relative humidity of 50%–60%. Tumour development was induced by streaking virulent *A. tumefaciens* strain C58 into a wound of 1.5 cm in length, scratched into the base of young 5‐cm‐long inflorescence stalks. Tumour tissue was harvested 28 days after infection using a scalpel and a binocular. Wounded, but uninfected, tumour‐free inflorescence stalk sections of the same age served as reference tissues.

### GS and camalexin analysis in *Arabidopsis* seedlings

Extraction and analysis of seedling GSs and camalexin were performed and modified as described previously (Glauser *et al*., 2012; Zandalinas *et al*., 2012). In total, 100 mg FW of *Arabidopsis* seedlings were homogenized and dissolved in 1 mL of 70% high‐performance liquid chromatography (HPLC)‐grade methanol containing 12.5 ng/μL sinalbin (4‐hydroxybenzyl GS) as an internal standard. The supernatants obtained were heated at 80 °C for 20 min and subjected to a UPLC‐Synapt G1 high‐definition mass spectrometry (HDMS) system (Waters, Taipei, Taiwan). GSs were separated on an Acquity CSH C_18_ column (length, 100 mm; 2.1 mm i.d.; 1.7 μm; Waters) at a flow rate of 400 μL/min. The GSs were eluted by solvent A (2% acetonitrile and 0.05% formic acid) and solvent B (100% acetonitrile and 0.05% formic acid) for 8 min in 1%–45% solvent B and 1 min in 45%–100% solvent B. The fractions were injected for MS analysis, and negative ion data were recorded in MS1 mode. The peak area was calculated by MassLynx software (Waters), and then normalized to nanomoles for GSs or micrograms for camalexin per gram FW. The GSs were quantified with the given references, including I3M for iGSs, 4MTB for methylthioalkyl GSs and 4MSOB for methylsulfinylalkyl GSs, purchased from AppliChem (Darmstadt, Germany). Camalexin was quantified with pure camalexin (Sigma‐Aldrich, St. Louis, MO, USA).

### GS and camalexin analysis in *Arabidopsis* inflorescence stalks

For GS analysis of infected *Arabidopsis* inflorescence stalks, 100 mg (FW) were lyophilized, thoroughly homogenized and extracted three times with 1 mL of 80% (v/v) methanol. For the first extraction step, benzyl GS (Phytoplan, Heidelberg, Germany) was added to each sample as internal standard. GSs were desulfonated as described previously (Agerbirk *et al*., 2001), and separated on a Grom‐Sil 80 ODS 7 pH column (length, 60 mm; 4 mm i.d.; 4 μm; Alltech) (Wuerzburg, Germany) by HPLC (Agilent 1200, Waldbronn, Germany; flow rate, 0.25 mL/min). The desulfo GSs were eluted as follows: 0.3 min in 0%–5% solvent A (water), 7 min with 1.2 min hold in 5%–95% solvent B (methanol) and 3.5 min in 95%–5% solvent B. Desulfo‐GSs were determined via UV diode array detection (229 nm), identified and quantified using particular response factors (aGSs, 1; iGSs, 0.26) (Gonzáles‐Megías and Müller, 2010).

Camalexin was extracted from lyophilized tissue (50 mg FW) by the addition of 400 μL of 85% methanol. The samples were thoroughly homogenized with a metal ball in a Mixer Mill 301 (Retsch, Haan, Germany) for 1.5 min at a frequency of 30 Hz. The extract was incubated at 42 °C for 60 min with addition of 0.3 μg/μL camalexin as an external standard. For the identification and quantification of camalexin, HPLC was applied as described by Mikkelsen *et al*. (2009).

### GS derived metabolites and camalexin treatment for transient transformation assays and *Agrobacterium* cell counts

The selected aGS‐ITCs (LKT Laboratories, St Paul, MN, USA) and camalexin were dissolved in DMSO, and I3M was dissolved in methanol. These compounds were added to the seedling co‐cultivation medium for *Agrobacterium* infection and GUS assays, as described above.

For the measurement of the viable *Agrobacterium* cell number, the bacterial cells (C58 strain carrying pBISNI) in co‐cultivation medium and associated with seedlings at 1 and 3 dpi were collected. Six seedlings per well were washed by 2 mL of double‐distilled H_2_O to remove unbound bacteria and ground by a mortar in 1 mL of 0.9% NaCl solution. The bacterial cells in medium or associated with seedlings were 10× serially diluted and then plated on 523 medium (Kado and Heskett, 1970) containing kanamycin, and incubated at 25 ºC for 2 days to obtain colony‐forming units (CFUs). The seedling‐associated *Agrobacterium* cell number was further normalized to the plant fresh weight.

### Myrosinase activity

Myrosinase activity was determined from 50–200 mg of frozen plant material, which was purified from internal substrate. Activity was measured by the photometric quantification of the released glucose from standardized amounts of externally added substrate according to the protocol developed by Travers‐Martin *et al*. (2008).

### Callus induction assay

Callus induction assay was performed and modified as described previously (Hwang and Gelvin, 2004). Col‐0 and the tested mutants were grown on half‐strength MS agar plates for 3 weeks, and the roots were cut into ∼1‐cm segments. About 60 root explants were transferred to agar plates containing callus induction medium (CIM), further incubated for 4 weeks, followed by counting of the number of developing calli and calculation of the rate of callus induction.

## Supporting information

Additional Supporting Information may be found in the online version of this article at the publisher's website:

**Fig. S1** T‐DNA‐encoded genes are expressed at detectable levels not before 24 h post‐infection (hpi) of *Arabidopsis* seedlings. (a–d) Seven‐day‐old Col‐0 seedlings were infected with *Agrobacterium tumefaciens* wild‐type C58 harbouring a β‐glucuronidase (GUS) construct, and analysed by GUS staining at 2–96 hpi. Representative images of infected seedlings at 24 hpi (b) or 48 hpi (c) of shoots and at 48 hpi of roots (d) are shown. (e) Percentage of shoots and roots with a GUS signal. (f–i) Col‐0 seedlings were transferred into timentin‐containing medium 2–72 h after infection with *A. tumefaciens* (infection time) and further incubated for another 24–94 h as indicated (recovery time), followed by GUS staining. Enlarged images show infected seedlings at 24 hpi (g) or 48 hpi (h) of shoots and at 24 hpi of roots (i). (j) Percentage of shoots and roots with a GUS signal. Scale bar: 1 cm (a, f); 1 mm (b–d, g–i).

**Fig. S2** Gene expression profiles of the glucosinolate (GS) and camalexin biosynthesis pathways in *Agrobacterium*‐infected inflorescence stalks. Biosynthesis pathways of indole glucosinolate (iGS) and camalexin (a) and of aliphatic glucosinolate (aGS) (b). Orange squares mark iGSs (a) and aGSs (b). The three squares next to the aGS pathway (b) present the fold changes (inoculated versus non‐inoculated) of key genes at 3 h post‐infection (hpi) (left), 6 days post‐infection (dpi) (middle) and 35 dpi (right). Differentially transcribed genes of four replicates are shown in red for up‐regulation and green for down‐regulation [false discovery rate (FDR), *P* < 0.05] as outlined in Lee *et al*. (2009), and the fold changes are presented as log_2_ values.

**Fig. S3** Glucosinolate (GS) profiles of *Agrobacterium*‐infected *Arabidopsis* seedlings at 3 days post‐infection (dpi). Mock (a) and infected (b) Col‐0 seedlings were collected at 3 dpi for ultra‐pressure liquid chromatography‐mass spectrometry (UPLC‐MS) analysis. The numbers marked above the peaks indicate the GS compound listed in Table S5, and sinalbin was used as an internal standard (IS). The peak area was calculated by MassLynx software (Waters, Taipei, Taiwan), and quantified by specific references. Concentrations of indole GSs (iGSs) (c) and aliphatic GSs (aGSs) (d) for mock (black) and *Agrobacterium*‐infected (white) seedlings. Asterisks indicate significant changes compared with the mock sample (Student's *t*‐test, *P < 0.05, *n* = 3). FW, fresh weight.

**Fig. S4** Effects of the glucosinolate (GS)‐derived metabolites and camalexin on β‐glucuronidase (GUS) enzyme activity. The recombinant GUS protein was incubated with indol‐3‐ylmethylglucosinolate (I3M) (a), aliphatic glucosinolate‐isothiocyanates (aGS‐ITCs) (b) and camalexin (c) for the assay of GUS enzymatic activity. The results are presented as the mean ± standard error of the mean (SEM) from three independent experiments (*n* = 9), and significant differences from the control groups methanol (MeOH) and dimethylsulfoxide (DMSO) are indicated [one‐way analysis of variance (ANOVA) with Dunnett's test, **P < 0.01, ***P < 0.001].

**Fig. S5** Viable *Agrobacterium* cell numbers in co‐cultivation medium and associated with the plant on indol‐3‐ylmethylglucosinolate (I3M) (a, b), aliphatic glucosinolate‐isothiocyanate (aGS‐ITC) (c, d) and camalexin (e, f) treatment at 1 and 3 days post‐infection (dpi). *Agrobacterium* colony‐forming units (CFU) obtained from six seedlings with similar size per well (*in planta*) (a, c, e) and from the medium per well (in medium) (b, d, f) at 1 and 3 dpi. *Agrobacterium* cell numbers associated with the seedlings were normalized to the plant fresh weight. Results are presented as the mean ± standard error of the mean (SEM) and asterisks indicate significant changes compared with the control groups methanol (MeOH) and dimethylsulfoxide (DMSO) [one‐way analysis of variance (ANOVA) with Dunnett's test, *P < 0.05, **P < 0.01, ***P < 0.001].

**Fig. S6** Root callus induction assays in the mutants lacking glucosinolates (GSs) and/or camalexin (a) and under camalexin‐treated conditions (b). (a) Root explants from 3‐week‐old Col‐0 and mutant plants were incubated on callus induction medium plates for 4 weeks. (b) Col‐0 root explants were incubated on callus induction medium plates containing different concentrations of camalexin for 4 weeks. The number of root explants producing callus was counted under a dissection microscope. Results are presented as the mean ± standard error of the mean (SEM) from three experiments (*n* ≥ 8), and asterisks indicate significant changes compared with the control Col‐0 or dimethylsulfoxide (DMSO) treatment [one‐way analysis of variance (ANOVA) with Dunnett's test, **P < 0.01, ***P < 0.001].

**Table S1** The enriched gene ontology (GO) items in shoots of C58‐infected seedlings at 2 h post‐infection (hpi).

**Table S2** The enriched gene ontology (GO) items in roots of C58‐infected seedlings at 2 h post‐infection (hpi).

**Table S3** The enriched gene ontology (GO) items in shoots of C58‐infected seedlings at 24 h post‐infection (hpi).

**Table S4** The enriched gene ontology (GO) items in roots of C58‐infected seedlings at 24 h post‐infection (hpi).

**Table S5** The detected glucosinolates and camalexin in *Arabidopsis* seedlings.

**Datasheet S1** The *Arabidopsis* gene list of *Agrobacterium*‐responsive genes in shoots and roots at 2 and 24 h post‐infection (hpi).

**Datasheet S2** Expression of the *Arabidopsis* Col‐0 genes involved in glucosinolate and camalexin biosynthesis, as well as glucosinolate hydrolysis.

## References

[mpp12672-bib-0001] Agerbirk, N. , Petersen, B.L. , Olsen, C.E. , Halkier, B.A. and Nielsen, J.K. (2001) 1,4‐Dimethoxyglucobrassicin in *Barbarea* and 4‐hydroxyglucobrassicin in *Arabidopsis* and *Brassica* . J. Agric. Food Chem. 49, 1502–1507. 11312886 10.1021/jf001256r

[mpp12672-bib-0002] Ahuja, I. , Kissen, R. and Bones, A.M. (2012) Phytoalexins in defense against pathogens. Trends Plant Sci. 17, 73–90. 22209038 10.1016/j.tplants.2011.11.002

[mpp12672-bib-0003] Aires, A. , Mota, V.R. , Saavedra, M.J. , Monteiro, A.A. , Simões, M. , Rosa, E.A.S. and Bennett, R.N. (2009) Initial in vitro evaluations of the antibacterial activities of glucosinolate enzymatic hydrolysis products against plant pathogenic bacteria. J. Appl. Microbiol. 106, 2096–2105. 19291239 10.1111/j.1365-2672.2009.04181.x

[mpp12672-bib-0004] Alexa, A. , Rahnenführer, J. and Lengauer, T. (2006) Improved scoring of functional groups from gene expression data by decorrelating GO graph structure. Bioinformatics, 22, 1600–1607. 16606683 10.1093/bioinformatics/btl140

[mpp12672-bib-0006] Bednarek, P. (2012) Sulfur‐containing secondary metabolites from *Arabidopsis thaliana* and other Brassicaceae with function in plant immunity. Chembiochem, 13, 1846–1859. 22807086 10.1002/cbic.201200086

[mpp12672-bib-0007] Bednarek, P. , Pislewska‐Bednarek, M. , Svatos, A. , Schneider, B. , Doubsky, J. , Mansurova, M. , Humphry, M. , Consonni, C. , Panstruga, R. , Sanchez‐Vallet, A. , Molina, A. and Schulze‐Lefert, P. (2009) A glucosinolate metabolism pathway in living plant cells mediates broad‐spectrum antifungal defense. Science, 323, 101–106. 19095900 10.1126/science.1163732

[mpp12672-bib-0008] Beekwilder, J. , van Leeuwen, W. , van Dam, N.M. , Bertossi, M. , Grandi, V. , Mizzi, L. , Soloviev, M. , Szabados, L. , Molthoff, J.W. , Schipper, B. , Verbocht, H. , de Vos, R.C.H. , Morandini, P. , Aarts, M.G.M. and Bovy, A. (2008) The impact of the absence of aliphatic glucosinolates on insect herbivory in *Arabidopsis* . PLoS One, 3, e2068. 18446225 10.1371/journal.pone.0002068 PMC2323576

[mpp12672-bib-0009] Boerjan, W. , Cervera, M.T. , Delarue, M. , Beeckman, T. , Dewitte, W. , Bellini, C. , Caboche, M. , Van Onckelen, H. , Van Montagu, M. and Inzé, D. (1995) Superroot, a recessive mutation in *Arabidopsis*, confers auxin overproduction. Plant Cell, 7, 1405–1419. 8589625 10.1105/tpc.7.9.1405 PMC160963

[mpp12672-bib-0010] Brader, G. , Mikkelsen, M.D. , Halkier, B.A. and Palva, E.T. (2006) Altering glucosinolate profiles modulates disease resistance in plants. Plant J. 46, 758–767. 16709192 10.1111/j.1365-313X.2006.02743.x

[mpp12672-bib-0011] Britton, M.T. , Escobar, M.A. and Dandekar, A.M. (2008) The oncogenes of *Agrobacterium tumefaciens* and *Agrobacterium rhizogenes* In: Agrobacterium: From Biology to Biotechnology (Tzfira T. and Citovsky V., eds.), pp. 523–563. New York, NY: Springer.

[mpp12672-bib-0012] Chen, S. , Petersen, B.L. , Olsen, C.E. , Schulz, A. and Halkier, B.A. (2001) Long‐distance phloem transport of glucosinolates in *Arabidopsis* . Plant Physiol. 127, 194–201. 11553747 10.1104/pp.127.1.194 PMC117975

[mpp12672-bib-0013] Clay, N.K. , Adio, A.M. , Denoux, C. , Jander, G. and Ausubel, F.M. (2009) Glucosinolate metabolites required for an *Arabidopsis* innate immune response. Science, 323, 95–101. 19095898 10.1126/science.1164627 PMC2630859

[mpp12672-bib-0014] Fan, J. , Crooks, C. , Creissen, G. , Hill, L. , Fairhurst, S. , Doerner, P. and Lamb, C. (2011) Pseudomonas sax genes overcome aliphatic isothiocyanate‐mediated non‐host resistance in *Arabidopsis* . Science, 331, 1185–1188. 21385714 10.1126/science.1199707

[mpp12672-bib-0015] Gaspar, Y.M. , Nam, J. , Schultz, C.J. , Lee, L.Y. , Gilson, P.R. , Gelvin, S.B. and Bacic, A . (2004) Characterization of the *Arabidopsis* lysine‐rich arabinogalactan‐protein AtAGP17 mutant (rat1) that results in a decreased efficiency of *Agrobacterium* transformation. Plant Physiol. 135, 2162–2171. 15286287 10.1104/pp.104.045542 PMC520787

[mpp12672-bib-0016] Gelvin, S.B. (2010) Plant proteins involved in *Agrobacterium*‐mediated genetic transformation. Annu. Rev. Phytopathol. 48, 45–68. 20337518 10.1146/annurev-phyto-080508-081852

[mpp12672-bib-0017] Glauser, G. , Schweizer, F. , Turlings, T.C. and Reymond, P. (2012) Rapid profiling of intact glucosinolates in *Arabidopsis* leaves by UHPLC‐QTOFMS using a charged surface hybrid column. Phytochem. Anal. 23, 520–528. 22323091 10.1002/pca.2350

[mpp12672-bib-0018] Glazebrook, J. and Ausubel, F.M. (1994) Isolation of phytoalexin‐deficient mutants of *Arabidopsis thaliana* and characterization of their interactions with bacterial pathogens. Proc. Natl. Acad. Sci. USA, 91, 8955–8959. 8090752 10.1073/pnas.91.19.8955 PMC44725

[mpp12672-bib-0019] Gohlke, J. and Deeken, R. (2014) Plant responses to *Agrobacterium tumefaciens* and crown gall development. Front Plant Sci. 5, 155. 24795740 10.3389/fpls.2014.00155 PMC4006022

[mpp12672-bib-0020] Gonzáles‐Megías, A. and Müller, C. (2010) Root herbivores and detritivores shape above‐ground multitrophic assemblage through plant‐mediated effects. J. Anim. Ecol. 79, 923–931. 20302605 10.1111/j.1365-2656.2010.01681.x

[mpp12672-bib-0021] Halkier, B.A. and Gershenzon, J. (2006) Biology and biochemistry of glucosinolates. Annu. Rev. Plant Biol. 57, 303–333. 16669764 10.1146/annurev.arplant.57.032905.105228

[mpp12672-bib-0022] Hwang, H.H. and Gelvin, S.B. (2004) Plant proteins that interact with VirB2, the *Agrobacterium tumefaciens* pilin protein, mediate plant transformation. Plant Cell, 16, 3148–3167. 15494553 10.1105/tpc.104.026476 PMC527204

[mpp12672-bib-0023] Hwang, H.H. , Wang, M.H. , Lee, Y.L. , Tsai, Y.L. , Li, Y.H. , Yang, F.J. , Liao, Y.C. , Lin, S.K. Lai, E.M. (2010) *Agrobacterium*‐produced and exogenous cytokinin‐modulated *Agrobacterium*‐mediated plant transformation. Mol. Plant Pathol. 11, 677–690. 20696005 10.1111/j.1364-3703.2010.00637.x PMC6640272

[mpp12672-bib-0024] Hwang, H.‐H. , Yang, F.‐J. , Cheng, T.‐F. , Chen, Y.‐C. , Lee, Y.‐L. , Tsai, Y.‐L. and Lai, E.‐M. (2013) The Tzs protein and exogenous cytokinin affect virulence gene expression and bacterial growth of *Agrobacterium tumefaciens* . Phytopathology, 103, 888–899. 23593941 10.1094/PHYTO-01-13-0020-R

[mpp12672-bib-0025] Hwang, H.H. , Yu, M. and Lai, E.M. (2017) *Agrobacterium*‐mediated plant transformation: biology and applications. Arabidopsis Book, 15, e0186. 31068763 10.1199/tab.0186 PMC6501860

[mpp12672-bib-0026] Kado, C.I. and Heskett, M.G. (1970) Selective media for isolation of *Agrobacterium*, *Corynebacterium*, *Erwinia*, *Pseudomonas*, and *Xanthomonas* . Phytopathology, 60, 969–976. 5469886 10.1094/phyto-60-969

[mpp12672-bib-0027] Kutacek, M. and Rovenska, J. (1991) Auxin synthesis in *Agrobacterium tumefaciens* and *A. tumefaciens*‐transformed plant‐tissue. Plant Growth Regul. 10, 313–327.

[mpp12672-bib-0028] Lee, C.‐W. , Efetova, M. , Engelmann, J.C. , Kramell, R. , Wasternack, C. , Ludwig‐Muller, J. , Hedrich, R. and Deeken, R. (2009) *Agrobacterium tumefaciens* promotes tumor induction by modulating pathogen defense in *Arabidopsis thaliana* . Plant Cell, 21, 2948–2962. 19794116 10.1105/tpc.108.064576 PMC2768927

[mpp12672-bib-0029] Lin, W.D. , Chen, Y.C. , Ho, J.M. and Hsiao, C.D. (2006) GOBU: toward an integration interface for biological objects. J. Inform. Sci. Eng. 22, 19–29.

[mpp12672-bib-0030] Lipka, V. , Dittgen, J. , Bednarek, P. , Bhat, R. , Wiermer, M. , Stein, M. , Landtag, J. , Brandt, W. , Rosahl, S. , Scheel, D. , Llorente, F. , Molina, A. , Parker, J. , Somerville, S. and Schulze‐Lefert, P. (2005) Pre‐ and postinvasion defenses both contribute to nonhost resistance in *Arabidopsis* . Science, 310, 1180–1183. 16293760 10.1126/science.1119409

[mpp12672-bib-0031] Malka, S.K. and Cheng, Y.F. (2017) Possible interactions between the biosynthetic pathways of indole glucosinolate and auxin. Front. Plant Sci. 8, 2131. 29312389 10.3389/fpls.2017.02131 PMC5735125

[mpp12672-bib-0032] Mikkelsen, M.D. , Fuller, V.L. , Hansen, B.G. , Nafisi, M. , Olsen, C.E. , Nielsen, H.B. and Halkier, B.A. (2009) Controlled indole‐3‐acetaldoxime production through ethanol‐induced expression of CYP79B2. Planta, 229, 1209–1217. 19263076 10.1007/s00425-009-0907-5

[mpp12672-bib-0034] Narasimhulu, S.B. , Deng, X.B. , Sarria, R. and Gelvin, S.B. (1996) Early transcription of *Agrobacterium* T‐DNA genes in tobacco and maize. Plant Cell, 8, 873–886. 8672885 10.1105/tpc.8.5.873 PMC161145

[mpp12672-bib-0035] Pieterse, C.M. , Leon‐Reyes, A. , Van der Ent, S. and Van Wees, S.C. (2009) Networking by small‐molecule hormones in plant immunity. Nat. Chem. Biol. 5, 308–316. 19377457 10.1038/nchembio.164

[mpp12672-bib-0036] Pitzschke, A. (2013) *Agrobacterium* infection and plant defense‐transformation success hangs by a thread. Front. Plant Sci. 4, 519. 24391655 10.3389/fpls.2013.00519 PMC3866890

[mpp12672-bib-0037] Regier, D.A. and Morris, R.O. (1982) Secretion of trans‐zeatin by *Agrobacterium tumefaciens* – a function determined by the nopaline Ti plasmid. Biochem. Biophys. Res. Commun. 104, 1560–1566. 7073755 10.1016/0006-291x(82)91429-2

[mpp12672-bib-0038] Rogers, E.E. , Glazebrook, J. and Ausubel, F.N. (1996) Mode of action of the *Arabidopsis thaliana* phytoalexin camalexin and its role in *Arabidopsis*–pathogen interactions. Mol. Plant–Microbe Interact. 9, 748–757. 8870273 10.1094/mpmi-9-0748

[mpp12672-bib-0039] Salinas, J. and Sánchez‐Serrano, J.J. (2006) Arabidopsis Protocols. Totowa, NJ: Humana Press.

[mpp12672-bib-0040] Sardesai, N. , Lee, L.‐Y. , Chen, H. , Yi, H. , Olbricht, G.R. , Stirnberg, A. , Jeffries, J. , Xiong, K. , Doerge, R.W. and Gelvin, S.B. (2013) Cytokinins secreted by *Agrobacterium* promote transformation by repressing a plant Myb transcription factor. Sci. Signal. 6, ra100. 24255177 10.1126/scisignal.2004518

[mpp12672-bib-0041] Shlezinger, N. , Minz, A. , Gur, Y. , Hatam, I. , Dagdas, Y.F. , Talbot, N.J. and Sharon, A. (2011) Anti‐apoptotic machinery protects the necrotrophic fungus *Botrytis cinerea* from host‐induced apoptotic‐like cell death during plant infection. PLoS Pathog. 7, e1002185. 21876671 10.1371/journal.ppat.1002185 PMC3158046

[mpp12672-bib-0042] Stachel, S.E. , Messens, E. , Van Montagu, M. and Zambryski, P. (1985) Identification of the signal molecules produced by wounded plant‐cells that activate T‐DNA transfer in *Agrobacterium tumefaciens* . Nature, 318, 624–629.

[mpp12672-bib-0043] Stepanova, A.N. , Yun, J. , Robles, L.M. , Novak, O. , He, W. , Guo, H. , Ljung, K. and Alonso, J.M. (2011) The *Arabidopsis* YUCCA1 flavin monooxygenase functions in the indole‐3‐pyruvic acid branch of auxin biosynthesis. Plant Cell, 23, 3961–3973. 22108406 10.1105/tpc.111.088047 PMC3246335

[mpp12672-bib-0044] Stotz, H.U. , Sawada, Y. , Shimada, Y. , Hirai, M.Y. , Sasaki, E. , Krischke, M. , Brown, P.D. , Saito, K. and Kamiya, Y. (2011) Role of camalexin, indole glucosinolates, and side chain modification of glucosinolate‐derived isothiocyanates in defense of *Arabidopsis* against *Sclerotinia sclerotiorum* . Plant J. 67, 81–93. 21418358 10.1111/j.1365-313X.2011.04578.x

[mpp12672-bib-0045] Sugawara, S. , Hishiyama, S. , Jikumaru, Y. , Hanada, A. , Nishimura, T. , Koshiba, T. , Zhao, Y. , Kamiya, Y. and Kasahara, H. (2009) Biochemical analyses of indole‐3‐acetaldoxime‐dependent auxin biosynthesis in *Arabidopsis* . Proc. Natl. Acad. Sci. USA, 106, 5430–5435. 19279202 10.1073/pnas.0811226106 PMC2664063

[mpp12672-bib-0046] Travers‐Martin, N. , Kuhlmann, F. and Müller, C. (2008) Revised determination of free and complexed myrosinase activities in plant extracts. Plant Physiol. Biochem. 46, 506–516. 18395461 10.1016/j.plaphy.2008.02.008

[mpp12672-bib-0047] Wittstock, U. and Burow, M. (2010) Glucosinolate breakdown in *Arabidopsis*: mechanism, regulation and biological significance. Arabidopsis Book, 8, e0134. 22303260 10.1199/tab.0134 PMC3244901

[mpp12672-bib-0048] Wu, H.‐Y. , Liu, K.‐H. , Wang, Y.‐C. , Wu, J.‐F. , Chiu, W.‐L. , Chen, C.‐Y. , Wu, S.‐H. , Sheen, J. and Lai, E.‐M. (2014) AGROBEST: an efficient *Agrobacterium*‐mediated transient expression method for versatile gene function analyses in *Arabidopsis* seedlings. Plant Methods, 10, 19. 24987449 10.1186/1746-4811-10-19 PMC4076510

[mpp12672-bib-0049] Yan, X.F. and Chen, S.X. (2007) Regulation of plant glucosinolate metabolism. Planta, 226, 1343–1352. 17899172 10.1007/s00425-007-0627-7

[mpp12672-bib-0050] Yu, Y.B. and Yang, S.F. (1979) Auxin‐induced ethylene production and its inhibition by aminoethoxyvinylglycine and cobalt ion. Plant Physiol. 64, 1074–1077. 16661095 10.1104/pp.64.6.1074 PMC543194

[mpp12672-bib-0051] Yuan, Z.‐C. , Edlind, M.P. , Liu, P. , Saenkham, P. , Banta, L.M. , Wise, A.A. , Ronzone, E. , Binns, A.N. , Kerr, K. and Nester, E.W. (2007) The plant signal salicylic acid shuts down expression of the vir regulon and activates quormone‐quenching genes in *Agrobacterium* . Proc. Natl. Acad. Sci. USA, 104, 11 790–11 795. 10.1073/pnas.0704866104 PMC1905925 17606909

[mpp12672-bib-0052] Zandalinas, S.I. , Vives‐Peris, V. , Gómez‐Cadenas, A. and Arbona, V. (2012) A fast and precise method to identify indolic glucosinolates and camalexin in plants by combining mass spectrometric and biological information. J. Agric. Food Chem. 60, 8648–8658. 22870889 10.1021/jf302482y

[mpp12672-bib-0053] Zhao, Y. , Hull, A.K. , Gupta, N.R. , Goss, K.A. , Alonso, J. , Ecker, J.R. , Normanly, J. , Chory, J. and Celenza, J.L. (2002) Trp‐dependent auxin biosynthesis in *Arabidopsis*: involvement of cytochrome P450s CYP79B2 and CYP79B3. Genes Dev. 16, 3100–3112. 12464638 10.1101/gad.1035402 PMC187496

[mpp12672-bib-0054] Zipfel, C. , Kunze, G. , Chinchilla, D. , Caniard, A. , Jones, J.D.G. , Boller, T. and Felix, G. (2006) Perception of the bacterial PAMP EF‐Tu by the receptor EFR restricts *Agrobacterium*‐mediated transformation. Cell, 125, 749–760. 16713565 10.1016/j.cell.2006.03.037

