# Supplementary figures and images for: Differential roles of glucosinolates and camalexin at different stages of Agrobacterium‐mediated transformation
Source: Mol Plant Pathol. 2018 Apr 23;19(8):1956–70. doi: 10.1111/mpp.12672 (PMC6638096; doi:10.1111/mpp.12672)

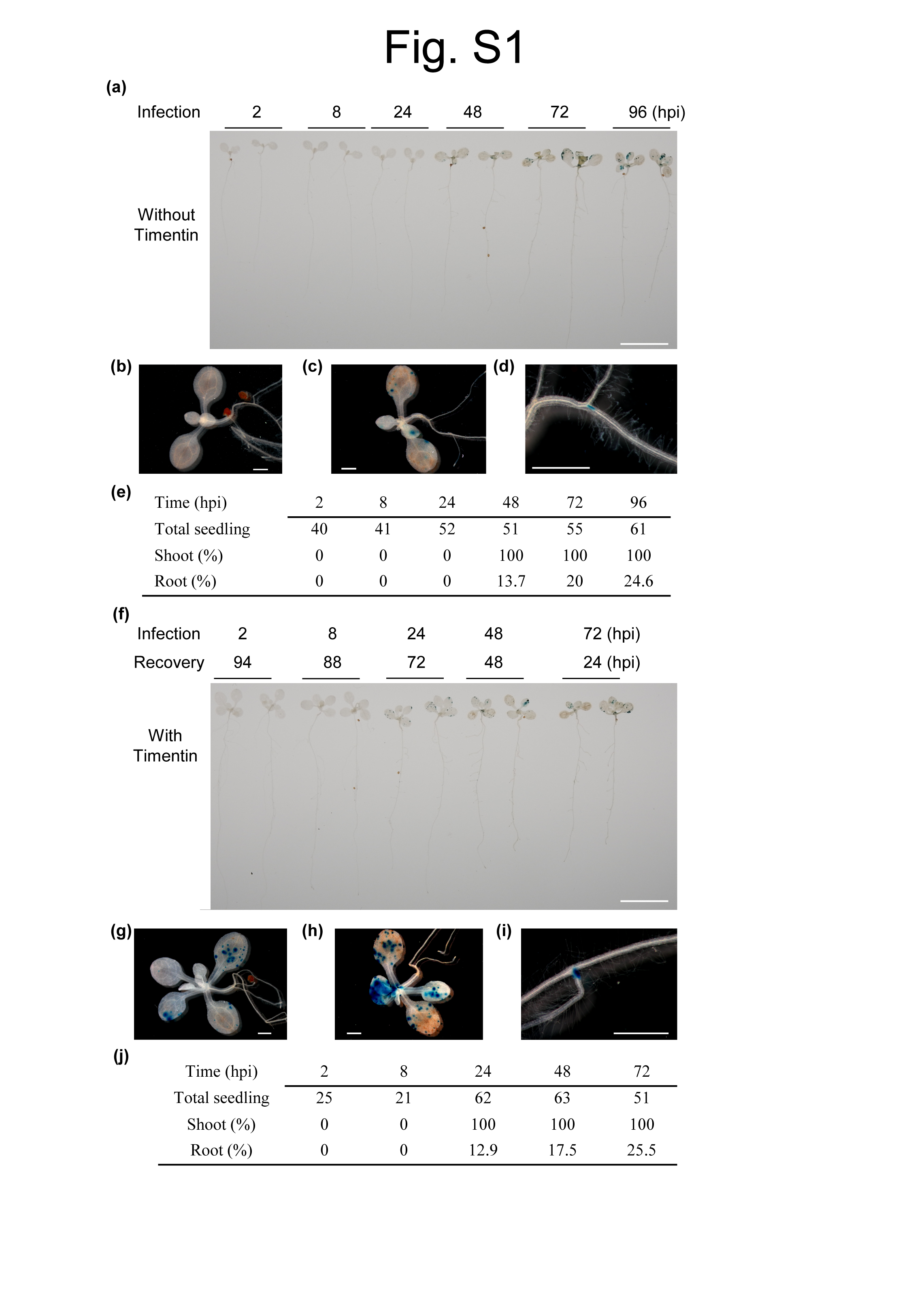

Supplement: Supplementary file 1 — Fig. S1 T‐DNA‐encoded genes are expressed at detectable levels not before 24 h post‐infection (hpi) of Arabidopsis seedlings. (a–d) Seven‐day‐old Col‐0 seedlings were infected with Agrobacterium tumefaciens wild‐type C58 harbouring a β‐glucuronidase (GUS) construct, and analysed by GUS staining at 2–96 hpi. Representative images of infected seedlings at 24 hpi (b) or 48 hpi (c) of shoots and at 48 hpi of roots (d) are shown. (e) Percentage of shoots and roots with a GUS signal. (f–i) Col‐0 seedlings were transferred into timentin‐containing medium 2–72 h after infection with A. tumefaciens (infection time) and further incubated for another 24–94 h as indicated (recovery time), followed by GUS staining. Enlarged images show infected seedlings at 24 hpi (g) or 48 hpi (h) of shoots and at 24 hpi of roots (i). (j) Percentage of shoots and roots with a GUS signal. Scale bar: 1 cm (a, f); 1 mm (b–d, g–i). [file MPP-19-1956-s001.tif]

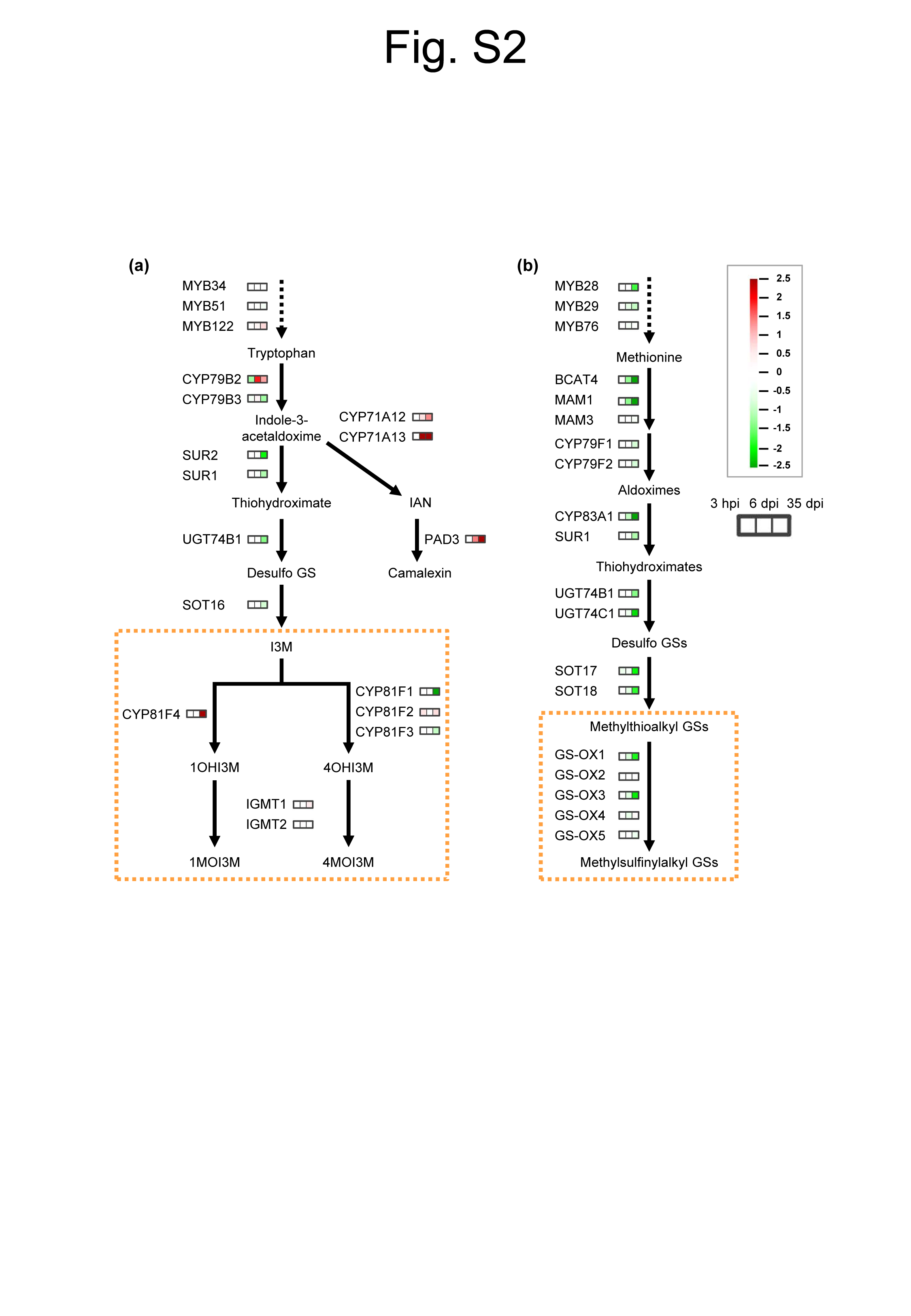

Supplement: Supplementary file 2 — Fig. S2 Gene expression profiles of the glucosinolate (GS) and camalexin biosynthesis pathways in Agrobacterium‐infected inflorescence stalks. Biosynthesis pathways of indole glucosinolate (iGS) and camalexin (a) and of aliphatic glucosinolate (aGS) (b). Orange squares mark iGSs (a) and aGSs (b). The three squares next to the aGS pathway (b) present the fold changes (inoculated versus non‐inoculated) of key genes at 3 h post‐infection (hpi) (left), 6 days post‐infection (dpi) (middle) and 35 dpi (right). Differentially transcribed genes of four replicates are shown in red for up‐regulation and green for down‐regulation [false discovery rate (FDR), P < 0.05] as outlined in Lee et al. (2009), and the fold changes are presented as log2 values. [file MPP-19-1956-s002.tif]

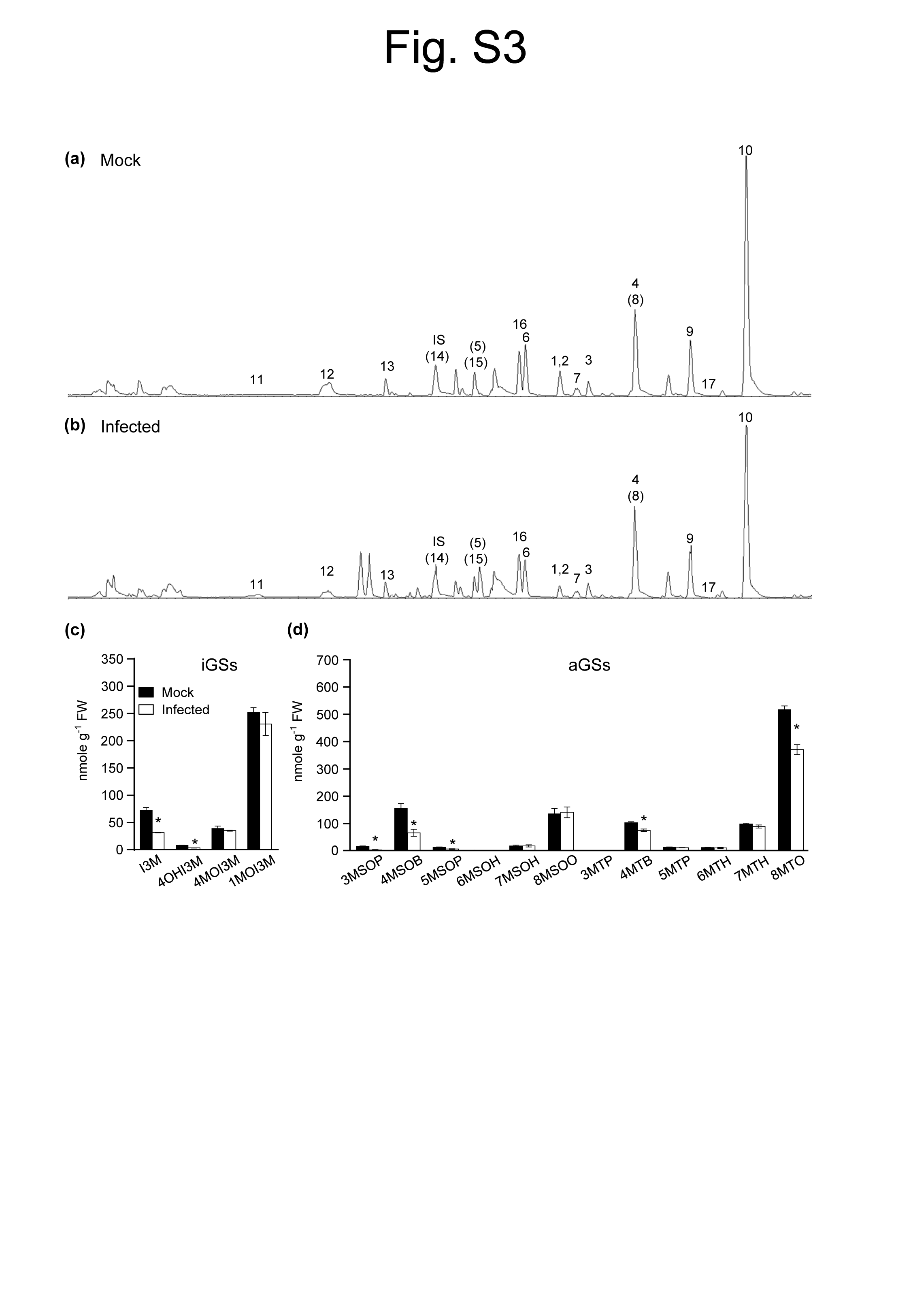

Supplement: Supplementary file 3 — Fig. S3 Glucosinolate (GS) profiles of Agrobacterium‐infected Arabidopsis seedlings at 3 days post‐infection (dpi). Mock (a) and infected (b) Col‐0 seedlings were collected at 3 dpi for ultra‐pressure liquid chromatography‐mass spectrometry (UPLC‐MS) analysis. The numbers marked above the peaks indicate the GS compound listed in Table S5, and sinalbin was used as an internal standard (IS). The peak area was calculated by MassLynx software (Waters, Taipei, Taiwan), and quantified by specific references. Concentrations of indole GSs (iGSs) (c) and aliphatic GSs (aGSs) (d) for mock (black) and Agrobacterium‐infected (white) seedlings. Asterisks indicate significant changes compared with the mock sample (Student's t‐test, *P < 0.05, n = 3). FW, fresh weight. [file MPP-19-1956-s003.tif]

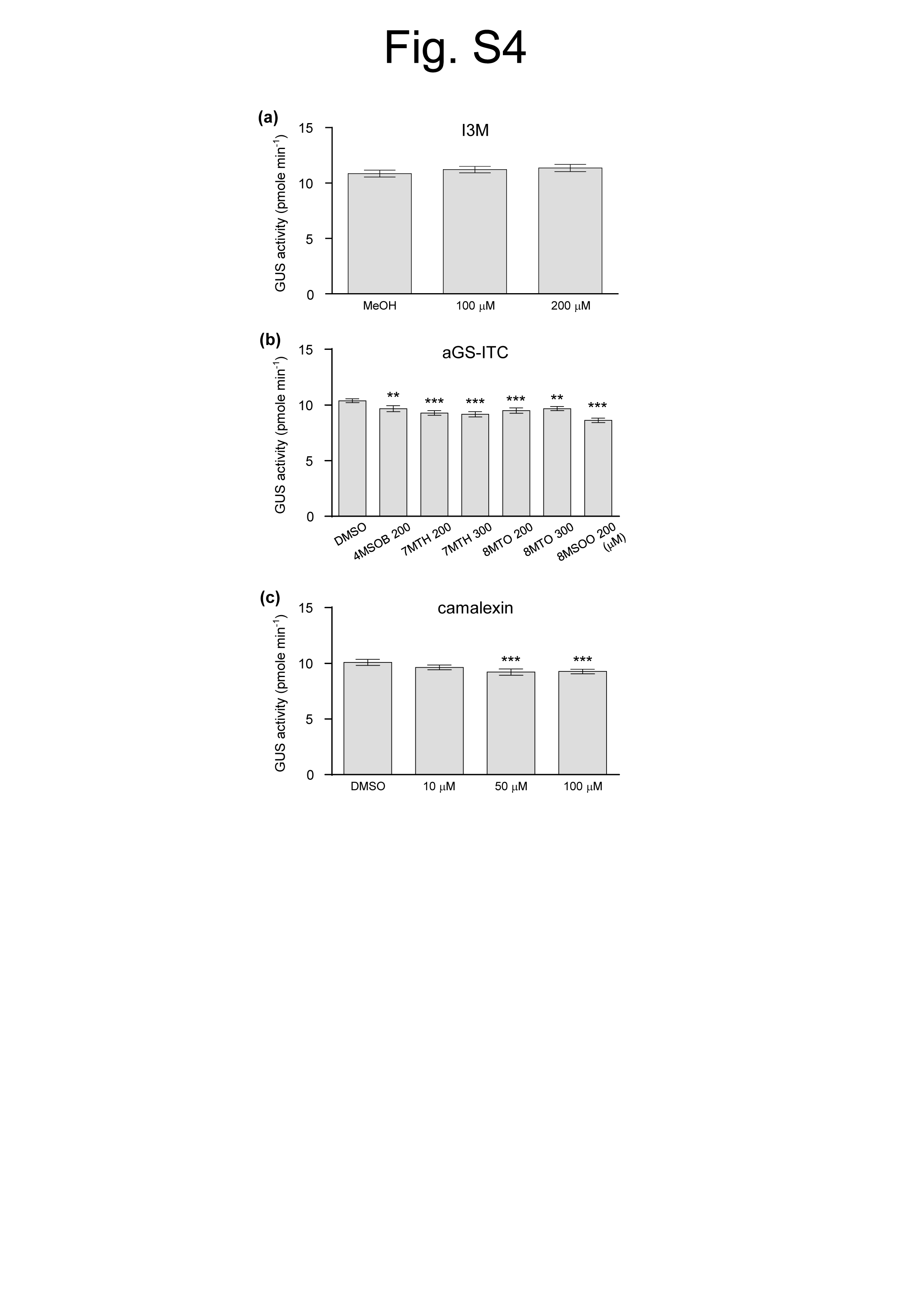

Supplement: Supplementary file 4 — Fig. S4 Effects of the glucosinolate (GS)‐derived metabolites and camalexin on β‐glucuronidase (GUS) enzyme activity. The recombinant GUS protein was incubated with indol‐3‐ylmethylglucosinolate (I3M) (a), aliphatic glucosinolate‐isothiocyanates (aGS‐ITCs) (b) and camalexin (c) for the assay of GUS enzymatic activity. The results are presented as the mean ± standard error of the mean (SEM) from three independent experiments (n = 9), and significant differences from the control groups methanol (MeOH) and dimethylsulfoxide (DMSO) are indicated [one‐way analysis of variance (ANOVA) with Dunnett's test, **P < 0.01, ***P < 0.001]. [file MPP-19-1956-s004.tif]

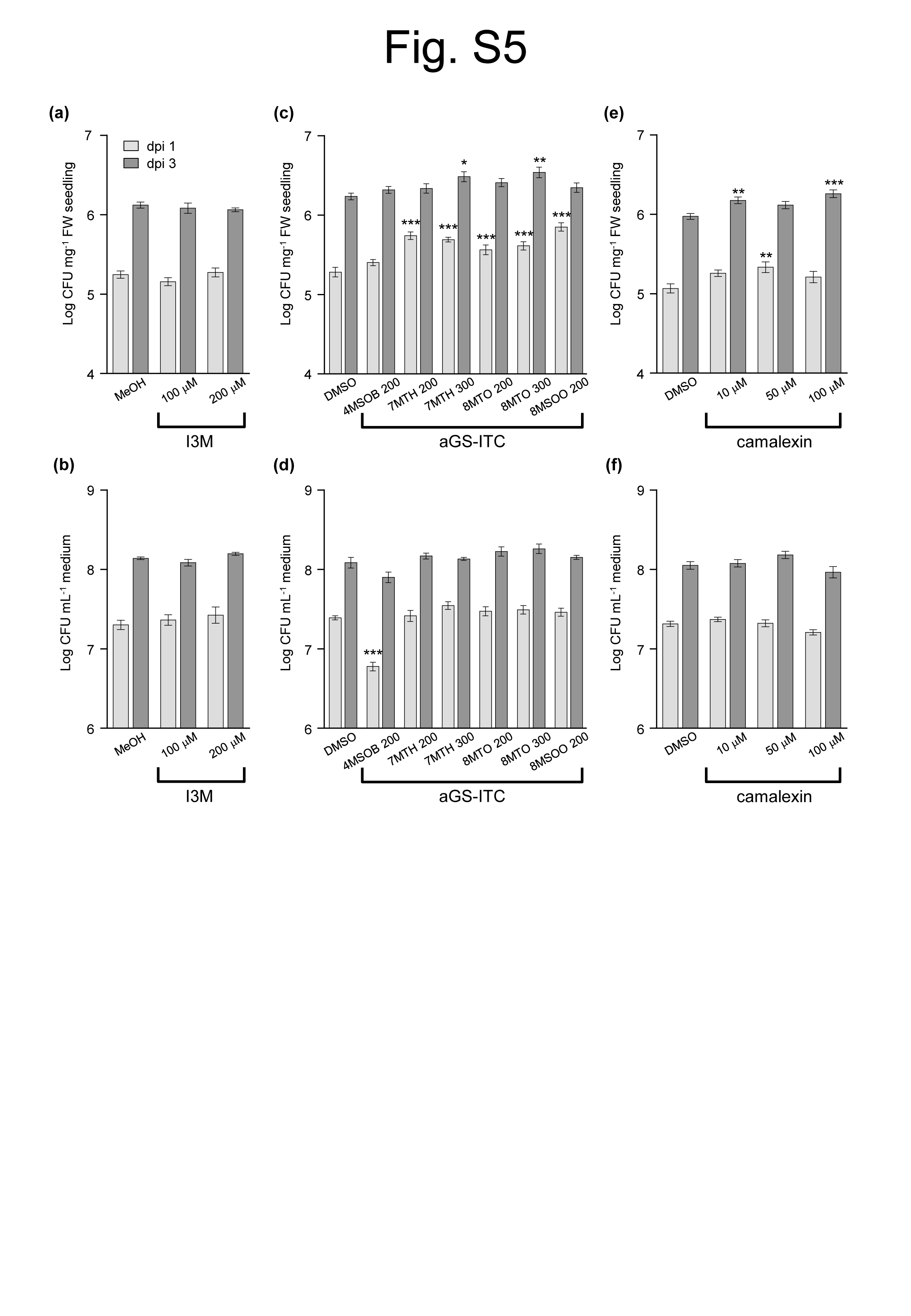

Supplement: Supplementary file 5 — Fig. S5 Viable Agrobacterium cell numbers in co‐cultivation medium and associated with the plant on indol‐3‐ylmethylglucosinolate (I3M) (a, b), aliphatic glucosinolate‐isothiocyanate (aGS‐ITC) (c, d) and camalexin (e, f) treatment at 1 and 3 days post‐infection (dpi). Agrobacterium colony‐forming units (CFU) obtained from six seedlings with similar size per well (in planta) (a, c, e) and from the medium per well (in medium) (b, d, f) at 1 and 3 dpi. Agrobacterium cell numbers associated with the seedlings were normalized to the plant fresh weight. Results are presented as the mean ± standard error of the mean (SEM) and asterisks indicate significant changes compared with the control groups methanol (MeOH) and dimethylsulfoxide (DMSO) [one‐way analysis of variance (ANOVA) with Dunnett's test, *P < 0.05, **P < 0.01, ***P < 0.001]. [file MPP-19-1956-s005.tif]

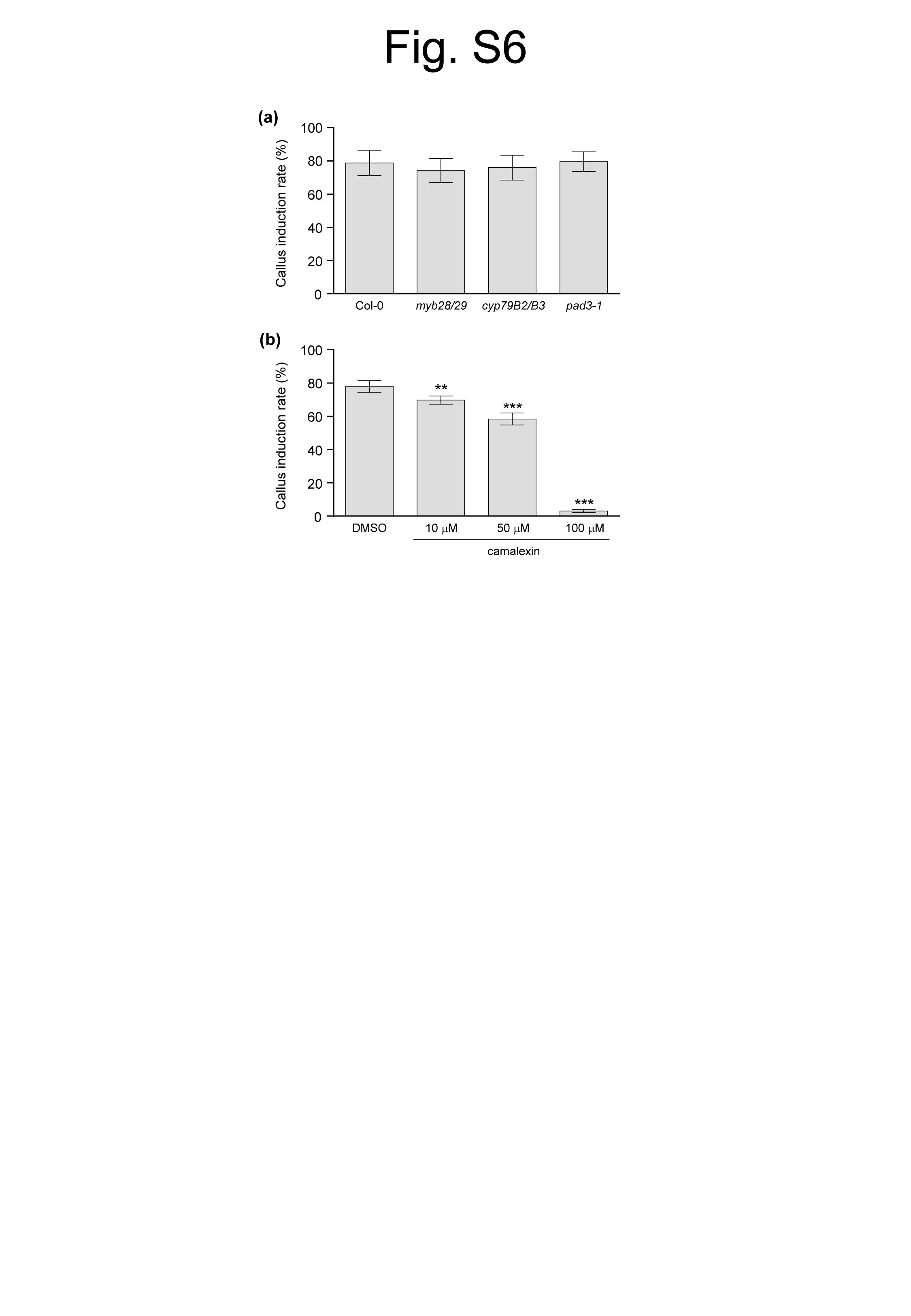

Supplement: Supplementary file 6 — Fig. S6 Root callus induction assays in the mutants lacking glucosinolates (GSs) and/or camalexin (a) and under camalexin‐treated conditions (b). (a) Root explants from 3‐week‐old Col‐0 and mutant plants were incubated on callus induction medium plates for 4 weeks. (b) Col‐0 root explants were incubated on callus induction medium plates containing different concentrations of camalexin for 4 weeks. The number of root explants producing callus was counted under a dissection microscope. Results are presented as the mean ± standard error of the mean (SEM) from three experiments (n ≥ 8), and asterisks indicate significant changes compared with the control Col‐0 or dimethylsulfoxide (DMSO) treatment [one‐way analysis of variance (ANOVA) with Dunnett's test, **P < 0.01, ***P < 0.001]. [file MPP-19-1956-s006.tif]
