# Supplementary material for: Differential roles of glucosinolates and camalexin at different stages of Agrobacterium‐mediated transformation
Source: Mol Plant Pathol. 2018 Apr 23;19(8):1956–70. doi: 10.1111/mpp.12672 (PMC6638096; doi:10.1111/mpp.12672)
Supplement: Supplementary file 7 — Table S1 The enriched gene ontology (GO) items in shoots of C58‐infected seedlings at 2 h post‐infection (hpi). [file MPP-19-1956-s007.docx]

Table S1: The enriched gene ontology (GO) items in shoots of C58-infected seedlings at 2 hours post infection (hpi)

| **GO Name** | **ID** | **Gene number** | | ***p* value** |
| --- | --- | --- | --- | --- |
|  |  | **Whole genome** | **DEG**^*^ |  |
| Cell wall modification |  |  |  |  |
| Plant-type cell wall loosening | GO:0009828 | 35 | 2 | 3.11E-04 |
| Plant-type cell wall modification | GO:0009827 | 47 | 3 | 8.89E-03 |
| Cellular reaction |  |  |  |  |
| Cellular phosphate ion homeostasis | GO:0030643 | 5 | 1 | 3.71E-03 |
| Development |  |  |  |  |
| Anatomical structure morphogenesis | GO:0009653 | 595 | 4 | 9.16E-04 |
| Cell growth | GO:0016049 | 290 | 3 | 1.27E-03 |
| Nutrient process |  |  |  |  |
| L-phenylalanine biosynthetic process | GO:0009094 | 8 | 1 | 5.94E-03 |
| Transport activity |  |  |  |  |
| Antibiotic transport | GO:0042891 | 2 | 1 | 1.49E-03 |

^*^ DEG: Differentially expressed genes of C58-infected Col-0 seedlings as shown in Datasheet S1.
