# Supplementary material for: Differential roles of glucosinolates and camalexin at different stages of Agrobacterium‐mediated transformation
Source: Mol Plant Pathol. 2018 Apr 23;19(8):1956–70. doi: 10.1111/mpp.12672 (PMC6638096; doi:10.1111/mpp.12672)
Supplement: Supplementary file 8 — Table S2 The enriched gene ontology (GO) items in roots of C58‐infected seedlings at 2 h post‐infection (hpi). [file MPP-19-1956-s008.docx]

Table S2: The enriched gene otology (GO) items in roots of C58-infected seedlings at 2 hours post infection (hpi)

| **GO Name** | **ID** | **Gene number** | | ***p* value** |
| --- | --- | --- | --- | --- |
|  |  | **Whole genome** | **DEG**^*^ |  |
| Cellular reaction |  |  |  |  |
| Oxidation-reduction process | GO:0055114 | 872 | 14 | 1.61E-06 |
| Development |  |  |  |  |
| Positive regulation of organ growth | GO:0046622 | 2 | 1 | 6.77E-03 |
| Hormone response |  |  |  |  |
| Cytokinin mediated signaling pathway | GO:0009736 | 42 | 4 | 1.27E-05 |
| Cytokinin metabolic process | GO:0009690 | 23 | 2 | 2.76E-03 |
| Jasmonic acid biosynthetic process | GO:0009695 | 24 | 2 | 3.00E-03 |
| Regulation of jasmonic acid mediated signaling pathway | GO:2000022 | 2 | 1 | 6.77E-03 |
| Response to abscisic acid stimulus | GO:0009737 | 359 | 5 | 7.70E-03 |
| Respond to other stimuli |  |  |  |  |
| Response to cold | GO:0009409 | 279 | 6 | 3.91E-04 |
| Response to water deprivation | GO:0009414 | 218 | 5 | 9.08E-04 |
| Response to salt stress | GO:0009651 | 416 | 6 | 3.01E-03 |
| Response to herbicide | GO:0009635 | 1 | 1 | 3.39E-03 |
| Secondary metabolism |  |  |  |  |
| Lignan biosynthetic process | GO:0009807 | 16 | 3 | 2.06E-05 |
| Thalianol metabolic process | GO:0080003 | 3 | 2 | 3.42E-05 |
| Pentacyclic triterpenoid biosynthetic process | GO:0019745 | 10 | 2 | 5.04E-04 |
| Tricyclic triterpenoid biosynthetic process | GO:0010263 | 2 | 1 | 6.77E-03 |
| Secondary metabolic process | GO:0019748 | 404 | 11 | 9.19E-03 |
| Transport activity |  |  |  |  |
| Regulation of iron ion transport | GO:0034756 | 1 | 1 | 3.39E-03 |

^*^ DEG: Differentially expressed genes of C58-infected Col-0 seedlings as shown in Datasheet S1.
