# Supplementary material for: Differential roles of glucosinolates and camalexin at different stages of Agrobacterium‐mediated transformation
Source: Mol Plant Pathol. 2018 Apr 23;19(8):1956–70. doi: 10.1111/mpp.12672 (PMC6638096; doi:10.1111/mpp.12672)
Supplement: Supplementary file 9 — Table S3 The enriched gene ontology (GO) items in shoots of C58‐infected seedlings at 24 h post‐infection (hpi). [file MPP-19-1956-s009.docx]

Table S3: The enriched gene ontology GO items in shoots of C58-infected seedlings at 24 hours post infection (hpi)

| **GO Name** | **ID** | **Gene number** | | ***p* value** |
| --- | --- | --- | --- | --- |
|  |  | **Whole genome** | **DEG**^*^ |  |
| Cell wall modification |  |  |  |  |
| Cell wall macromolecule catabolic process | GO:0016998 | 22 | 4 | 9.23E-05 |
| Cellular reaction |  |  |  |  |
| Protein phosphorylation | GO:0006468 | 931 | 34 | 1.51E-09 |
| Recognition of pollen | GO:0048544 | 33 | 4 | 4.69E-04 |
| Cell surface receptor linked signaling pathway | GO:0007166 | 151 | 6 | 6.88E-03 |
| Cellular aromatic compound metabolic process | GO:0006725 | 375 | 10 | 9.50E-03 |
| Defense response |  |  |  |  |
| Plant-type hypersensitive response | GO:0009626 | 42 | 8 | 1.78E-08 |
| Defense response to bacterium | GO:0042742 | 214 | 15 | 1.98E-08 |
| Response to wounding | GO:0009611 | 153 | 11 | 1.21E-06 |
| Defense response signaling pathway, resistance gene-independent | GO:0010204 | 10 | 3 | 1.52E-04 |
| Defense response to fungus, incompatible interaction | GO:0009817 | 41 | 4 | 1.08E-03 |
| Negative regulation of defense response | GO:0031348 | 19 | 3 | 1.14E-03 |
| Response to chitin | GO:0010200 | 127 | 6 | 2.98E-03 |
| Regulation of systemic acquired resistance | GO:0010112 | 9 | 2 | 4.18E-03 |
| Response to reactive oxygen species | GO:0000302 | 67 | 4 | 6.55E-03 |
| Immune effector process | GO:0002252 | 36 | 3 | 7.33E-03 |
| Response to molecule of bacterial origin | GO:0002237 | 12 | 2 | 7.50E-03 |
| Systemic acquired resistance, salicylic acid mediated signaling pathway | GO:0009862 | 12 | 2 | 7.50E-03 |
| Hormone response |  |  |  |  |
| Response to jasmonic acid stimulus | GO:0009753 | 163 | 10 | 1.51E-05 |
| Response to abscisic acid stimulus | GO:0009737 | 359 | 13 | 2.09E-04 |
| Light response |  |  |  |  |
| Response to absence of light | GO:0009646 | 16 | 3 | 6.77E-04 |
| Nutrient process |  |  |  |  |
| Leucine biosynthetic process | GO:0009098 | 9 | 3 | 1.08E-04 |
| Xyloglucan metabolic process | GO:0010411 | 6 | 2 | 1.78E-03 |
| Carboxylic acid catabolic process | GO:0046395 | 97 | 5 | 4.54E-03 |
| Respond to other stimuli |  |  |  |  |
| Response to karrikin | GO:0080167 | 129 | 8 | 9.97E-05 |
| Cellular response to cold | GO:0070417 | 10 | 3 | 1.52E-04 |
| Response to ozone | GO:0010193 | 27 | 4 | 2.12E-04 |
| Cellular response to sulfur starvation | GO:0010438 | 7 | 2 | 2.47E-03 |
| Cellular response to salt stress | GO:0071472 | 10 | 2 | 5.19E-03 |
| Cellular response to water deprivation | GO:0042631 | 12 | 2 | 7.50E-03 |
| Response to fructose stimulus | GO:0009750 | 13 | 2 | 8.80E-03 |
| Secondary metabolism |  |  |  |  |
| Regulation of glucosinolate biosynthetic process | GO:0010439 | 6 | 2 | 1.78E-03 |
| Transport activity |  |  |  |  |
| Lipid transport | GO:0006869 | 147 | 9 | 4.08E-05 |
| Amino acid import | GO:0043090 | 5 | 2 | 1.20E-03 |
| Ion transport | GO:0006811 | 418 | 12 | 2.63E-03 |
| Drug transmembrane transport | GO:0006855 | 63 | 4 | 5.27E-03 |

^*^ DEG: Differentially expressed genes of C58-infected Col-0 seedlings as shown in Datasheet S1.
