# Supplementary material for: Differential roles of glucosinolates and camalexin at different stages of Agrobacterium‐mediated transformation
Source: Mol Plant Pathol. 2018 Apr 23;19(8):1956–70. doi: 10.1111/mpp.12672 (PMC6638096; doi:10.1111/mpp.12672)
Supplement: Supplementary file 10 — Table S4 The enriched gene ontology (GO) items in roots of C58‐infected seedlings at 24 h post‐infection (hpi). [file MPP-19-1956-s010.docx]

Table S4: The enriched gene ontology (GO) items in roots of C58-infected seedlings at 24 hours post infection (hpi)

| **GO Name** | **ID** | **Gene number** | | ***p* value** |
| --- | --- | --- | --- | --- |
|  |  | **Whole genome** | **DEG**^*^ |  |
| Cell wall modification |  |  |  |  |
| Suberin biosynthetic process | GO:0010345 | 8 | 4 | 1.21E-04 |
| Cell wall macromolecule catabolic process | GO:0016998 | 22 | 5 | 1.12E-03 |
| Plant-type cell wall loosening | GO:0009828 | 35 | 6 | 1.73E-03 |
| Cellular reaction |  |  |  |  |
| Oxidation-reduction process | GO:0055114 | 872 | 81 | 4.14E-13 |
| Toxin catabolic process | GO:0009407 | 46 | 9 | 4.38E-05 |
| Two-component signal transduction system (phosphorelay) | GO:0000160 | 78 | 11 | 1.49E-04 |
| Defense response |  |  |  |  |
| Defense response to fungus | GO:0050832 | 132 | 14 | 4.38E-04 |
| Response to wounding | GO:0009611 | 153 | 17 | 6.27E-05 |
| Response to bacterium | GO:0009617 | 274 | 19 | 7.62E-03 |
| Defense response to insect | GO:0002213 | 12 | 3 | 8.90E-03 |
| Development |  |  |  |  |
| Primary root development | GO:0080022 | 16 | 4 | 2.47E-03 |
| Lateral root development | GO:0048527 | 65 | 8 | 2.84E-03 |
| Root hair elongation | GO:0048767 | 42 | 6 | 4.47E-03 |
| Aging | GO:0007568 | 96 | 9 | 9.82E-03 |
| Hormone response |  |  |  |  |
| Cytokinin catabolic process | GO:0009823 | 7 | 5 | 1.43E-06 |
| Response to ethylene stimulus | GO:0009723 | 153 | 19 | 4.77E-06 |
| Response to abscisic acid stimulus | GO:0009737 | 359 | 31 | 1.58E-05 |
| Cytokinin mediated signaling pathway | GO:0009736 | 42 | 8 | 1.42E-04 |
| Indoleacetic acid biosynthetic process | GO:0009684 | 10 | 4 | 3.40E-04 |
| Response to jasmonic acid stimulus | GO:0009753 | 163 | 15 | 1.23E-03 |
| Auxin homeostasis | GO:0010252 | 19 | 4 | 4.80E-03 |
| Cytokinin biosynthetic process | GO:0009691 | 10 | 3 | 5.13E-03 |
| Response to auxin stimulus | GO:0009733 | 288 | 20 | 6.20E-03 |
| Light response |  |  |  |  |
| Response to red light | GO:0010114 | 55 | 16 | 9.94E-11 |
| Response to far red light | GO:0010218 | 43 | 11 | 3.65E-07 |
| Response to blue light | GO:0009637 | 52 | 10 | 1.94E-05 |
| Chlorophyll biosynthetic process | GO:0015995 | 36 | 7 | 3.23E-04 |
| Nonphotochemical quenching | GO:0010196 | 6 | 3 | 9.57E-04 |
| Response to absence of light | GO:0009646 | 16 | 4 | 2.47E-03 |
| Photosystem II repair | GO:0010206 | 8 | 3 | 2.53E-03 |
| Nutrient process |  |  |  |  |
| Trehalose biosynthetic process | GO:0005992 | 20 | 8 | 3.14E-07 |
| Leucine biosynthetic process | GO:0009098 | 9 | 5 | 8.04E-06 |
| Leucine catabolic process | GO:0006552 | 5 | 3 | 4.92E-04 |
| Very long-chain fatty acid metabolic process | GO:0000038 | 22 | 5 | 1.12E-03 |
| Alcohol biosynthetic process | GO:0046165 | 48 | 7 | 1.93E-03 |
| Glycogen metabolic process | GO:0005977 | 3 | 2 | 4.08E-03 |
| Cellular carbohydrate catabolic process | GO:0044275 | 155 | 13 | 5.59E-03 |
| Reductive pentose-phosphate cycle | GO:0019253 | 11 | 3 | 6.86E-03 |
| Hexose metabolic process | GO:0019318 | 112 | 10 | 9.32E-03 |
| Fatty acid biosynthetic process | GO:0006633 | 130 | 11 | 9.70E-03 |
| Respond to other stimuli |  |  |  |  |
| Response to oxidative stress | GO:0006979 | 278 | 40 | 3.09E-13 |
| Response to karrikin | GO:0080167 | 129 | 20 | 7.19E-08 |
| Response to nitrate | GO:0010167 | 20 | 5 | 7.02E-04 |
| Response to fructose stimulus | GO:0009750 | 13 | 4 | 1.06E-03 |
| Response to sucrose stimulus | GO:0009744 | 44 | 7 | 1.14E-03 |
| Positive gravitropism | GO:0009958 | 14 | 4 | 1.44E-03 |
| Hyperosmotic salinity response | GO:0042538 | 48 | 7 | 1.93E-03 |
| Cellular response to starvation | GO:0009267 | 109 | 11 | 2.58E-03 |
| Response to desiccation | GO:0009269 | 19 | 4 | 4.80E-03 |
| Response to metal ion | GO:0010038 | 446 | 28 | 5.65E-03 |
| Secondary metabolism |  |  |  |  |
| Glucosinolate biosynthetic process | GO:0019761 | 37 | 13 | 4.04E-10 |
| Fat-soluble vitamin biosynthetic process | GO:0042362 | 14 | 4 | 1.44E-03 |
| Indole glucosinolate metabolic process | GO:0042343 | 14 | 4 | 1.44E-03 |
| Lignan biosynthetic process | GO:0009807 | 16 | 4 | 2.47E-03 |
| Spermine biosynthetic process | GO:0006597 | 3 | 2 | 4.08E-03 |
| Transport activity |  |  |  |  |
| Acidic amino acid transport | GO:0015800 | 4 | 3 | 2.03E-04 |
| Hydrogen peroxide transmembrane transport | GO:0080170 | 5 | 3 | 4.92E-04 |
| Ammonium transport | GO:0015696 | 6 | 3 | 9.57E-04 |
| Protein import into chloroplast thylakoid membrane | GO:0045038 | 6 | 3 | 9.57E-04 |
| Phosphate transport | GO:0006817 | 13 | 4 | 1.06E-03 |
| Lipid transport | GO:0006869 | 147 | 13 | 3.58E-03 |

^*^ DEG: Differentially expressed genes of C58-infected Col-0 seedlings as shown in Datasheet S1.
