# Supplementary material for: Differential roles of glucosinolates and camalexin at different stages of Agrobacterium‐mediated transformation
Source: Mol Plant Pathol. 2018 Apr 23;19(8):1956–70. doi: 10.1111/mpp.12672 (PMC6638096; doi:10.1111/mpp.12672)
Supplement: Supplementary file 11 — Table S5 The detected glucosinolates and camalexin in Arabidopsis seedlings. [file MPP-19-1956-s011.docx]

Table S5: The detected glucosinolates in Col-0 seedlings, and sinalbin was used as an internal standard (IS).

| No. | Formula | *m/z* | Error  (ppm) | Systematic name (Abbreviation) | Common name |
| --- | --- | --- | --- | --- | --- |
| IS | C_14_H_18_NO_10_S_2_ | 424.0377 | 3.7 | 4-Hydroxybenzyl- | Sinalbin |
| Indole glucosinolate | | | | | |
| 1 | C_16_H_19_N_2_O_9_S_2_ | 447.0530 | 4.1 | Indol-3-ylmethyl- (I3M) | Glucobrassicin |
| 2 | C_16_H_19_N_2_O_10_S_2_ | 463.0486 | 3.3 | 4-Hydroxyindol-3-ylmethyl- (4OHI3M) | Hydroxyglucobrassicin |
| 3 | C_17_H_21_N_2_O_10_S_2_ | 477.0633 | 6.4 | 4-Methoxyindol-3-ylmethyl- (4MOI3M) | Methoxyglucobrassicin |
| 4 | C_17_H_21_N_2_O_10_S_2_ | 477.0632 | 3.1 | 1-Methoxyindol-3-ylmethyl- (1MOI3M) | Neoglucobrassicin |
| (Aliphatic) Methylthioalkyl glucosinolate | | | | | |
| 5 | C_11_H_20_NO_9_S_3_ | 406.0298 | 12 | 3-Methylthiopropyl- (3MTP) | Glucoiberverin |
| 6 | C_12_H_22_NO_9_S_3_ | 420.046 | 4.7 | 4-Methylthiobutyl- (4MTB) | Glucoerucin |
| 7 | C_13_H_24_NO_9_S_3_ | 434.0602 | 3.8 | 5-Methylthiopentyl- (5MTP) | Glucoberteroin |
| 8 | C_14_H_26_NO_9_S_3_ | 448.0760 | 10.5 | 6-Methylthiohexyl- (6MTH) | Glucolesquerellin |
| 9 | C_15_H_28_NO_9_S_3_ | 462.0922 | 6.2 | 7-Methylthioheptyl- (7MTH) |  |
| 10 | C_16_H_30_NO_9_S_3_ | 476.1075 | 5.4 | 8-Methylthiooctyl- (8MTO) |  |
| (Aliphatic) Methylsulfinylalkyl glucosinolate | | | | | |
| 11 | C_11_H_20_NO_10_S_3_ | 422.0248 | 6.5 | 3-Methylsulfinylpropyl- (3MSOP) | Glucoiberin |
| 12 | C_12_H_22_NO_10_S_3_ | 436.0402 | 3.4 | 4-Methylsulfinylbutyl- (4MSOB) | Glucoraphanin |
| 13 | C_13_H_24_NO_10_S_3_ | 450.0564 | 2.8 | 5-Methylsulfinylpentyl- (5MSOP) | Glucoalyssin |
| 14 | C_14_H_26_NO_10_S_3_ | 464.0725 | 7.9 | 6-Methylsulfinylhexyl- (6MSOH) | Glucohesperin |
| 15 | C_15_H_28_NO_10_S_3_ | 478.0878 | 3.7 | 7-Methylsulfinylheptyl- (7MSOH) | Glucoibarin |
| 16 | C_16_H_30_NO_10_S_3_ | 492.1036 | 3.4 | 8-Methylsulfinyloctyl- (8MSOO) | Glucohirsutin |
| Camalexin | | | | | |
| 17 | C_11_H_8_N_2_S | 199.0329 | 4.9 | 3-(1,3-thiazol-2-yl)-1H-indole | Camalexin |
